# Exploring the multifaceted role of obesity in breast cancer progression

**DOI:** 10.3389/fcell.2024.1408844

**Published:** 2024-07-08

**Authors:** Sooraj Kakkat, Prabhat Suman, Elba A. Turbat- Herrera, Seema Singh, Debanjan Chakroborty, Chandrani Sarkar

**Affiliations:** ^1^ Department of Pathology, University of South Alabama, Mobile, AL, United States; ^2^ Cancer Biology Program, Mitchell Cancer Institute, University of South Alabama, Mobile, AL, United States; ^3^ Department of Biochemistry and Molecular Biology, University of South Alabama, Mobile, AL, United States

**Keywords:** obesity, adipokines, breast cancer, pathogenesis, therapeutic resistance

## Abstract

Obesity is a multifaceted metabolic disorder characterized by excessive accumulation of adipose tissue. It is a well-established risk factor for the development and progression of breast cancer. Adipose tissue, which was once regarded solely as a passive energy storage depot, is now acknowledged as an active endocrine organ producing a plethora of bioactive molecules known as adipokines that contribute to the elevation of proinflammatory cytokines and estrogen production due to enhanced aromatase activity. In the context of breast cancer, the crosstalk between adipocytes and cancer cells within the adipose microenvironment exerts profound effects on tumor initiation, progression, and therapeutic resistance. Moreover, adipocytes can engage in direct interactions with breast cancer cells through physical contact and paracrine signaling, thereby facilitating cancer cell survival and invasion. This review endeavors to summarize the current understanding of the intricate interplay between adipocyte-associated factors and breast cancer progression. Furthermore, by discussing the different aspects of breast cancer that can be adversely affected by obesity, this review aims to shed light on potential avenues for new and novel therapeutic interventions.

## 1 Introduction

Breast cancer (BCa), caused by the uncontrolled proliferation of breast epithelial cells (Siegel et al., 2020), is the most frequently diagnosed cancer and the second leading cause of cancer-related death among women in the United States (US). American cancer society (ACS) estimates that there will be 310,720 new cases of BCa in women in 2024, with 42,250 patients dying due to the disease (Siegel et al., 2024). In recent years, alongside increased BCa incidence, the increasing numbers of overweight and obese people also pose as a significant health challenge in the US (Ward et al., 2019). Furthermore, obesity affects BCa carcinogenesis, progression, and clinical outcome, and approximately 70% of BCa patients in the US are at increased risk for disease recurrence and death due to obesity (Jiralerspong and Goodwin, 2016). Obesity results in worse disease free survival (DFS) and overall survival (OS) in all nonmetastatic BCa subtypes [hormone receptor positive/HER2 negative (HR + HER2−), HER2 positive (HER2+), and triple negative (TNBC)] (Lohmann et al., 2021). Furthermore, postmenopausal obese women with hormone receptor-positive (HR+) BCa have a higher chance of disease recurrence (Harborg et al., 2023). Reports further indicate that fat tissue also supports the metastatic cancer cells, which leads to a worse prognosis and accelerated tumor spread (Annett et al., 2020). Recent research advances have highlighted the importance of the interactions between cancer cells and fat cells or adipocytes in disease progression. Adipocytes secrete various bioactive molecules, collectively known as adipokines that, in addition to playing essential roles in energy homeostasis, inflammation, and immunity, influence tumor growth and progression through direct and indirect interactions with cancer cells. Thus, manipulating adipokines or interfering with lipid metabolism pathways may offer new strategies for therapeutic intervention in BCa. This review aims to explore the intricate relationship between BCa and obesity, shedding light on the role of adipocytes and adipocyte derived factors during the pathogenesis of BCa. Scientific literature databases, including PubMed, MEDLINE, Scopus, and Google Scholar, were searched for studies on obesity, BCa pathogenesis, and therapeutic resistance, and studies published between 1989 and 2024 were included.

## 2 Adipocytes in breast cancer

Adipocytes account for a large proportion of human breast tissues (Rybinska et al., 2020) and are responsible for storing energy in the form of triglycerides. They also play essential roles in metabolic regulation, endocrine signaling, and immune functions (Maurizi et al., 2018). During adipogenesis, the process of differentiation and maturation of pre-adipocytes into adipocytes, pre-adipocytes undergo a series of transcriptional and morphological changes under the influence of adipogenic transcription factors (e.g., peroxisome proliferator activated receptor gamma (PPAR-γ) and CCAAT enhancer binding protein alpha (C/EBP-α)) and other signaling molecules. This process ultimately leads to the accumulation of lipid droplets within the mature adipocytes (Moseti et al., 2016). Adipogenesis plays a crucial role in BCa progression as cancer cells locally infiltrate the surrounding adipose tissue, leading to the activation and transformation of adjacent adipocytes into cancer-associated adipocytes (CAAs) found within the tumor microenvironment (TME) in cancer patients (Dirat et al., 2011; Wang et al., 2012). This is advantageous for BCa cells (Yamaguchi et al., 2008) as although the adipocytes traditionally are passive cells involved only in energy storage and release, the CAAs (Rybinska et al., 2021) actively support tumor cell survival, growth, and metastatic dissemination by secreting various signaling molecules and proinflammatory cytokines such as interleukin-6 (IL-6) and tumor necrosis factor-alpha (TNF-alpha) (Wu et al., 2019; Rybinska et al., 2020). CAAs also provide energy to cancer cells by exhibiting lipolysis which releases metabolites (Nieman et al., 2011). Higher levels of IL-6 were reported in surrounding adipocytes when the tumor sizes were bigger (Chen et al., 2016). In addition, *in vitro* studies have revealed an altered adipocyte phenotype characterized by delipidation and reduced expressions of adipocyte markers associated with an activated state that features overexpression of proteases, including matrix metalloproteinase-11 and proinflammatory cytokines [interleukin (IL)-6, IL-1β] when adipocytes are cultured with cancer cells. The downregulation of adipocyte terminal differentiation marker gene expression such as PPAR-γ and C/EBP-α display CAAs as dedifferentiated phenotype (Dirat et al., 2011).

## 3 Adipokines and breast cancer

Increased adiposity results in higher levels of hormones like estrogen, insulin, and other adipokines that promote an environment conducive to BCa progression. The increase of the majority of circulating adipokines like, leptin, resistin, visfatin, osteopontin, apelin, and lipocalin has been linked with BCa progression. In contrast, reduced circulating levels of certain adipokines like adiponectin and iridine (also known as adipo-mycin) have been shown to play a protective role against it. This section will discuss the functions of some of these common adipokines in BCa. The functions have also been summarized in Table 1.

**TABLE 1:** Adipokines in breast cancer.

Adipokine	General function	Function in BCa	Mechanism of action	References
Leptin	Central nervous system-based regulation of appetite Plays an important role in early stage cancer stem cell (CSC) survival	• Improves cell development and survival • Promotes tumor cell proliferation, migration and invasion and inhibits apoptosis	• Activates JAK2-STAT3, MAPK and PI3K/AKT signaling pathway • Increases CYCLIN D1 expression and activates Stat3 signaling pathway • Activates ERK pathway and increases the proliferation and migration of BCa cells	Saxena et al. (2007); Kelesidis et al. (2010); Park and Scherer (2011); Saxena and Sharma (2013); Yuan et al. (2014); Park et al. (2017); Thiagarajan et al. (2017); Wang et al. (2018b); Sánchez-Jiménez et al. (2019); Lipsey et al. (2020); Park et al. (2022)
Adiponectin	Anti-inflammatory and insulin sensitizer	• Inhibits cell proliferation and angiogenesis • Reduces inflammation and modulate immunological reactions	• Upregulates phosphorylation of AMPK • Inhibits mTOR activation • Cell cycle arrest at G0-G1 • Low adiponectin promotes cell proliferation and MAPK activation	Wang et al. (2006); Ouchi and Walsh (2007); Mauro et al. (2014); Spyrou et al. (2018); Andò et al. (2019); Choi et al. (2020); Nehme et al. (2022)
Resistin	Stimulates the release of IL-6 and TNF from macrophages, which leads to insulin resistance and inflammation	• Associated with tumor development • Promotes the expression of vimentin and phosphorylates c-src, PP2A, PKCα, ezrin, radixin, and moesin • Regulates the expression of IL-6 and increases the proliferation and aggressive behavior of BCa cells	• AMPK/mTOR/ULK1 and JNK signaling pathways • Induction of EMT and stemness by TLR4/NF-κB/STAT3 signaling pathway • Activates STAT3 signaling pathway	Deshmukh et al. (2015); Lee et al. (2016); Wang et al. (2018a); Sudan et al. (2020)
Chemerin	Functions as a chemoattractant by binding to the immune cell-expressed chemerin/chemokine-like receptor (CMKLR-1)	• Associated with increased malignancy in breast tissues • Increased serum levels of chemerin associated with higher tumor grades and elevated expressions of the cell proliferation marker, Ki67	• Upregulates VEGF, MMP-7 and interleukin-6 expressions via phosphorylation of p38 and extracellular signal-regulated kinase1/2 MAPK	El-Sagheer et al. (2018); Kennedy and Davenport (2018); Kim et al. (2020); Song et al. (2021)
Visfatin	Catalyzes the conversion of nicotinamide to nicotinamide mononucleotide (NMN)	• Maintains tumor cell survival and growth • Overexpression of Visfatin in BCa has been associated with BCa pathogenesis	• Promotes the survival of macrophages via an IL-6/STAT3-dependent pathway • Visfatin-Notch1 axis contributes to breast tumor growth through the activation of the NF-κB pathway • Activates AKT/PI3K, and ERK/MAPK. • Produces lipids through the EGFR/PI3K/AKT/GSB3β/SREBP-1 signaling pathway	Park et al. (2014); Gholinejad et al. (2017); Dakroub et al. (2020); Rajput et al. (2023)
Osteopontin	Biomineralization, chemotaxis, inflammation, and cell stimulation by integrin activation	• Correlates with advanced stages, increased metastasis and poorer outcomes	• Controls the secretion levels of interleukin-10 (IL-10), interleukin-12 (IL-12), interleukin-3 (IL-3), interferon-γ (IFN-γ), integrin αvB3, nuclear factor kappa B (NF-kB), macrophage, and T cells, as well as CD44 receptors • Stimulates the JAK2/STAT3 signaling pathway • Increases HIF-1α via the PI3K/AKT pathway in BCa models	Lund et al. (2009); Behera et al. (2010); Shevde et al. (2010); Raja et al. (2014); Icer and Gezmen-Karadag (2018)
Apelin	Acts as a vasodilator, involved in the regulation of fluid balance in the body, anti-inflammatory properties, modulating immune responses	• Promotes tumor growth and metastasis • Promotes angiogenesis and modulates the tumor microenvironment	• Activates PI3K/AKT and extracellular signal-regulated kinase (ERK) activation in vascular smooth muscle cells to reduce apoptosis	Tang et al. (2007); Cui et al. (2010); Uribesalgo et al. (2019); Hu et al. (2021); Hu et al. (2022)
Lipocalin 2	Increases inflammatory responses and insulin resistance by secreting TNF from adipocytes	• Associated with pro-inflammatory effects • Promotes tumor cell invasion and metastasis	• Upregulates NF-κB, ERK and JAK-STAT signaling pathways	Wang et al. (2011); Linjawi et al. (2018); Jaberi et al. (2021)

### 3.1 Leptin

Leptin is predominantly an adipocyte-specific16 kDa peptide hormone. It acts through a transmembrane receptor called leptin receptor (Ob-R or LEPR), to regulate processes like food intake, appetite, energy homeostasis, and immune response. It plays an important role in early stage cancer stem cell (CSC) survival and stimulates cancer cells’ growth, invasion, and migration once the primary tumors are established. Moreover, it influences stromal cells, including fibroblasts, immune, and endothelial cells, to promote angiogenesis and inflammatory processes that support the tumor growth (Park and Scherer, 2011). Studies have demonstrated that leptin affects the growth of breast tumors both directly by interacting with breast tumor cells and indirectly by impacting different elements of the TME (Saxena and Sharma, 2013). Studies investigating the role of the leptin-axis manipulation on breast carcinogenesis using genetic loss-of-function or mutation or by dietary manipulation have demonstrated how the development of mammary cancers is dependent upon an intact leptin-axis as obese mice lacking leptin or leptin receptors did not develop tumors (Cleary et al., 2003; Cleary et al., 2004; Dogan et al., 2007).

Moreover, studies have also reported that both primary and metastatic BCa express higher levels of leptin and its receptor than noncancerous tissues (Sato et al., 1991). Meta-analysis and epidemiological studies indicate that high serum leptin positively correlates with the increased risk of BCa (Niu et al., 2013; Sanchez-Infantes et al., 2014), aggressive tumors, and poor patient outcomes (Pan et al., 2018). Ki-67 expression and lymph node metastases are linked with leptin and receptor expressions. It directly promotes tumor cell proliferation, migration and invasion, inhibits apoptosis and indirectly influences tissue sensitivity to insulin, inflammatory responses, and angiogenesis as it controls the expressions of several factors that are important for carcinogenesis, including cyclin D1, p53, survivin, E-Cadherin, vascular endothelial growth factor (VEGF) and VEGF receptor 2 (VEGFR2) (Sanchez-Infantes et al., 2014; Nepal et al., 2015). Furthermore, leptin was reported to promote epithelial-to-mesenchymal transition (EMT) and cancer stem cell enrichment. However, the spectrum of its action is presumably much more comprehensive, as several cells in the TME possess its receptors (Bowers et al., 2018). By stimulating interleukin-18 (IL-18) and interleukin-8 (IL-8) production from M2 macrophages, it mediates the crosstalk between cancer cells and other cells in the TME. Activating leptin signaling in BCa leads to concurrent activation of multiple oncogenic pathways such as the JAK2/STAT3 pathway, MAPK pathway, and the PI3K pathway. Activation of the JAK2/STAT3 pathway increases BCa cell growth, promotes EMT, and thereby, invasion and migration of cancer cells, cancer cell stemness and chemoresistance (Park et al., 2017; Thiagarajan et al., 2017; Wang T. et al., 2018; Buonaiuto et al., 2022). Activation of the ERK pathway increases the proliferation and migration of BCa cells (Yuan et al., 2014). The PI3K/AKT pathway was reported to participate in IL-8 (Wang et al., 2015) and pyruvate kinase M2 (PKM2) upregulation mediated by leptin (Wei et al., 2016) leading to EMT. Reports further indicate that leptin via the PI3K/AKT/SREBP2 signaling pathway upregulates acetyl-CoA acetyltransferase 2 (ACAT2) to promote the proliferation, migration, and invasion of BCa cells (Huang et al., 2017). It was also reported that leptin mediated increased proliferation of BCa cells involved a significant increase in CYCLIN D1 expression and activation of Stat3 signaling pathway (Saxena et al., 2007). In addition, Leptin has also been reported to regulate exosome synthesis and release from various BCa cells, including MDA-MB-231 (TNBC) and MCF-7 (ER+) cells (Giordano et al., 2019).

### 3.2 Adiponectin

Adiponectin is a 30 kDa glycoprotein mainly secreted by white adipose tissue that acts through two classical receptors, AdipoR1, which is mainly expressed in skeletal muscles, synovial fibroblasts, and endothelial cells and AdipoR2, which is primarily expressed in the liver and a potential third receptor, T-cadherin or CDH13. Adiponectin plays a protective role in the development of metabolic disorders related to obesity (Nehme et al., 2022). A higher risk of BCa has been linked to low serum adiponectin levels, while high serum levels may act protectively against it (Georgiou et al., 2016). The results from thirty-one studies examining the relationship between adiponectin and BCa risk showed that BCa patients had significantly lower serum adiponectin levels (Gu et al., 2018). Data from multiple meta-analyses also indicate that postmenopausal BCa patients have significantly lower serum adiponectin levels (Yoon et al., 2019). Lower adiponectin levels were also noted in BCa-associated fat than in fat near benign lesions (Wang et al., 2014). Furthermore, as seen in obese mice and humans, decreased adiponectin expression leads to increased AdipoR2 gene methylation, inhibiting adiponectin binding and function. High expressions of AdipoR2 in BCa tissues strongly and favorably correlated with higher vascular and lymphovascular invasion (Parida et al., 2019). CAAs also exhibit significant upregulation of AdipoRs mRNA (Yao and He, 2021). Adipocyte DNA methylation of the adiponectin promoter region is linked to decreased adiponectin levels in obesity. However, it has also been noted that AdipoR1 methylation is unaffected by obesity (Naimo et al., 2023). Adiponectin was reported to exhibit anticancer properties (anti-proliferative, anti-migratory, and pro-apoptotic) (Choi et al., 2018). The anti-proliferation effect of adiponectin is manifested by cell cycle arrest and the pro-apoptotic effect by regulating B-cell lymphoma 2 (Bcl-2), Bcl-2 homologous antagonist killer (Bak), and p53. In addition, adiponectin also exhibits anti-angiogenic properties and regulates inflammatory cytokines (Chen and Wang, 2011). Adiponectin interacts with adiponectin receptors (AdipoR1 and AdipoR2) and activates AMPK (Choi et al., 2018) and PI3K/AKT (Groeneveld et al., 2016). Interestingly, studies also suggest a paradoxical role of adiponectin in BCa, where it has been demonstrated to stimulate the proliferation of ERα+ MCF-7 cells and inhibit the proliferation of ERα- MDA-MB-231 cells. A rapid activation of MAPK phosphorylation only in ERα+ MCF-7 was cited as a reason for increased proliferation (Mauro et al., 2014).

As adiponectin and leptin have antagonistic effects, the ratio of adiponectin to leptin is commonly used to assess their effect in BCa (Cleary et al., 2009). Both adiponectin and leptin are synthesized in adipose tissue. The serum levels of these adipokines are controlled by body weight/fat and dietary factors. The body fat condition affects leptin and adiponectin levels in an opposing manner. The levels of serum leptin increase with increasing body fat, and the levels of adiponectin decrease with increasing fat, resulting in higher adiponectin: leptin ratio in normal individuals compared to obese individuals (Grossmann and Cleary, 2012).

### 3.3 Resistin

Resistin encoded by the RETN gene on chromosome 19 (19p13.2) (Papakonstantinou et al., 2022) was first identified in 2001. It is a 108-amino acid cysteine-rich adipose tissue-specific peptide hormone, often referred to as adipose tissue-specific secretory factor (ADSF) (Patel et al., 2003). It has three isoforms: resistin-like molecule-α, β, and γ (RELM-α, β, and γ), and was initially identified in adipose tissue in mice (Steppan et al., 2001). It is involved in physiological processes like energy metabolism and pathological conditions like insulin resistance, inflammation, and obesity related diseases. Clinical evidence indicates that high serum resistin levels in BCa patients are associated with larger tumor sizes, advanced stages, lymph node metastases, and reduced survival rates (Lee et al., 2012; Wang C.-H. et al., 2018). Elevated resistin in BCa promotes the invasion and metastasis of cancer cells by inducing phosphorylation of c-Src, PP2A, PKCα, ezrin, radixin, and moesin. The concentration of intracellular calcium is elevated by resistin, and the activation of Src by resistin is inhibited when intracellular calcium is chelated (Lee et al., 2016). Adenylyl cyclase associated protein 1 (CAP1) was the first identified binding partner for human resistin. Clinical research indicates the association between CAP1 expression, promotion of malignant tumors, and poor survival rates in BCa patients (Bergqvist et al., 2020). CAP1 gene silencing decreases the ability of BCa cells to proliferate and migrate *in vitro* (Wang et al., 2021). Resistin promotes EMT by altering the transcriptional program and upregulating the expressions of mesenchymal markers such as snail family transcriptional repressor 1 (SNAI1), snail family transcriptional repressor 2 (SLUG or SNAI2), zinc finger E-box binding homeobox 1 (ZEB1), twist family BHLH transcription factor 1 (TWIST1), fibronectin, and vimentin and downregulating the expressions of epithelial markers (E-cadherin and claudin-1) by Cap1-dependent and independent mechanisms (Avtanski et al., 2019). A comparative study revealed a favorable link between the serum levels of resistin and IL-6. Treating BCa cells with resistin increased the production of IL-6. Additionally, resistin increased the expression and phosphorylation of the signal transducer and activator of transcription 3 (STAT3), while IL-6-neutralizing antibody therapy of BCa cells before resistin stimulation eliminated STAT3 phosphorylation (Deshmukh et al., 2015). It was also reported that higher serum levels of the pro-inflammatory cytokine resistin in cancer patients (Sudan et al., 2020) promoted the stemness of BCa cells (Deshmukh et al., 2017). A report indicated that resistin promotes stemness of BCa cells and EMT principally via toll-like receptor 4 (TLR-4) by activation of STAT3 and nuclear factor kappa-light-chain-enhancer of activated B cells (NF-κB) pathways (Wang C.-H. et al., 2018).

### 3.4 Chemerin

Chemerin, a 14 kDa protein, is the product of the tazarotene-induced gene 2 (TIG2) and is also referred to as retinoid acid receptor responder protein 2 (RARRES2). It mediates functions like promotion of angiogenesis, cell proliferation, and mobilization by acting through three receptors, namely, chemokine-like receptor 1 (CMKLR1), G-protein-coupled receptor 1 (GPR1), and C-C motif chemokine receptor-like 2 (CCRL2) (Kaur et al., 2010; Song et al., 2021; Yu et al., 2022). It was reported that malignant breast tissues express more chemerin than the surrounding normal breast tissues. This elevated expression is linked to poor prognosis (El-Sagheer et al., 2018). Additionally, it was reported that BCa patients show increased serum levels of chemerin, which are associated with higher tumor grades and elevated expressions Ki67. Therefore, it was suggested that the serum levels of chemerin could be used as a supplementary parameter for BCa prognosis (Song et al., 2021). The mechanism by which chemerin could increase cell proliferation is, however, unclear and could be attributed to the proangiogenic functions of chemerin (Song et al., 2021).

### 3.5 Visfatin

Visfatin, identified in 2005, is a large 52 kD protein with enzymatic activity. It is also known as pre-B-cell colony-enhancing factor 1 (PBEF1) and nicotinamide phosphoribosyl-transferase (NAMPT) and is encoded by the NAMPT gene located on chromosome 7q22.2 (Luhn et al., 2013; Ilhan et al., 2015). It has two forms: the intracellular (iNampt) form, which is involved in NAD salvage pathways and the extracellular form (eNampt), which is associated with intracellular signaling cascades (Dakroub et al., 2020). Overexpression of visfatin in BCa has been associated with BCa pathogenesis and decreased overall and disease-free patient survival (Lee et al., 2011; Christodoulatos et al., 2019; Wang et al., 2021; Ghaneialvar et al., 2022). Serum visfatin level was reported to be higher in postmenopausal BCa than control (Dalamaga et al., 2011; Assiri and Kamel, 2016). The findings of a case-control study with 204 patients indicate that serum visfatin/nicotinamide phosphoribosyl-transferase levels, independent of adiponectin, leptin levels, and other metabolic parameters, are related to postmenopausal BCa risk (Dalamaga et al., 2011). In premenopausal BCa patients, however, elevated serum visfatin levels were not always reported (Assiri et al., 2015). Interestingly, while some studies have reported that ER-, PR- and HER2- BCa patients exhibit higher serum visfatin levels (Dalamaga et al., 2012; Assiri and Kamel, 2016; Wang et al., 2021), absence of any discernible association between receptor and growth factor status and visfatin levels have also been reported (Hung et al., 2016; Zhu et al., 2016; Wang et al., 2021). A preclinical study further showed that high visfatin level increases the survival rate of BCa cells by upregulating the mRNA levels of cyclin D1 and cyclin-dependent kinase 2 (CDK2) and by activating the STAT3 pathway through the ABL proto-oncogene 1 (c-Abl) activator (Hung et al., 2016). Another study reported that visfatin regulates BCa cell proliferation and DNA synthesis rate by promoting the G1-to-S phase cell cycle transition and increases angiogenesis and metastasis by upregulation of VEGF and matrix metalloproteinase expressions (Kim et al., 2010). Other studies have reported the role of visfatin in the activation of the NF-κB (Park et al., 2014), AKT/PI3K, and ERK/MAPK (Gholinejad et al., 2017) pathways, upregulation of survivin and inhibition of caspase-mediated PARP activation (Gholinejad et al., 2017) in BCa resulting in accelerated cell proliferation and reduced apoptosis. In addition, an indirect method of activation by visfatin whereby it primes adipose-derived stem cells (ADSCs) and thereby upregulates growth differentiation factor 15 (GDF15) to activate AKT has also been reported (Huang et al., 2019). A recent study indicates that visfatin enhances lipid production and accumulation in MCF seven via EGFR/PI3K/AKT/GSB3β/SREBP-1 signaling pathway that promotes cell survival and proliferation. Restricting the synthesis and accumulation of fatty acids caused by visfatin through the suppression of SREBP-1 or other upstream pathway regulators could, therefore, regulate the survival and proliferation of BCa cells (Rajput et al., 2023).

### 3.6 Osteopontin

Osteopontin (OPN), sometimes referred to as bone/sialoprotein I (BSP-1), is a major adipokine that originates from the bone since it was initially shown to be a component of the ECM of normal bone tissue. However, it is also expressed in various other tissues, including adipocytes and the placenta. Its functions include biomineralization, chemotaxis, inflammation, and cell stimulation by integrin activation. According to recent research, OPN has been linked to numerous diseases, including diabetes, obesity, and several cancers, including BCa (Martinetti et al., 2004). Higher levels of OPN have been reported in BCa patients, which correlates with advanced stages, increased metastasis, and poorer outcomes. OPN promotes angiogenesis, immune evasion, and premetastatic niche formation (Icer and Gezmen-Karadag, 2018). A meta-analysis of 1567 BCa patients revealed a substantial association between increased OPN levels and a higher overall mortality rate. Furthermore, the expression of its splice variant-c was found to be even more strongly linked to a poorer prognosis, suggesting that OPN and OPN-c could be used as potential future prognostic indicators for BCa (Hao et al., 2016). In a randomized clinical trial with a large cohort of BCa patients treated with adjuvant chemotherapy, high expression of OPN mRNA was linked to decreased disease-free and overall survival (Psyrri et al., 2017). In addition, adverse outcomes were associated with both high baseline serum OPN and OPN gene mutation. Interestingly, triple negative BCa (TNBC) subtypes were reported to have frequent OPN gene mutations (Elbaiomy et al., 2020). OPN increases the expression of EMT-related transcription factors, such as Twist, Snail, and Slug, in BCa (Kothari et al., 2016). It has been reported to elevate hypoxia-inducible factor 1-alpha (HIF-1α) through the PI3K/AKT pathway (Raja et al., 2014), and a natural diterpenoid lactone, andrographolide, was reported to suppress BCa growth by downregulating OPN expression and PI3K/AKT signaling pathway (Kumar et al., 2012). Furthermore, reports also indicate that OPN can stimulate the JAK2/STAT3 pathway, promoting the expression of Bcl2 and the cell cycle regulator Cyclin D1, resulting in apoptosis resistant BCa cells with increased growth rate (Behera et al., 2010).

### 3.7 Apelin

Obesity increases the levels of Apelin, an evolutionarily conserved peptide that acts through the G protein-coupled Apelin receptor (APLNR). Preproapelin, the precursor form of Apelin, consists of 77 amino acids. Distinct mature isoforms such as apelin-36, apelin-17, apelin-13, or pyr apelin-13, a more stable pyroglutaminated form of apelin 13 that is the main circulating form of the adipokine, are formed upon successive cleavages (Watts, 1990; Habata et al., 1999). Human and mouse adipocytes exhibit a direct control of apelin production by insulin, which is linked to the activation of MAPK, phosphatidylinositol 3-kinase, and protein kinase C. Insulin and plasma apelin levels were both considerably higher in obese individuals, indicating that insulin-mediated apelin regulation may have an impact on apelin blood concentrations (Boucher et al., 2005).

Several correlation studies have recently indicated that apelin plays a role in tumor progression, with advanced stages of BCa showing high apelin expressions. BCa patients with elevated apelin levels tend to have worse prognoses and apelin expression is associated with lymph node metastasis, tumor size, stage, and histological type (Hu et al., 2022). In retrospective exploratory research, increased tumor apelin expression and obesity were independently linked to lower pathological complete response rates in 62 early BCa patients treated with taxane and anthracycline-based neoadjuvant chemotherapy. This observation lends credence to the idea that obesity and certain adipokines may influence the response to chemotherapy in addition to their involvement in the development of BCa (Gourgue et al., 2021). It was reported that subcutaneous adipose tissue and tumors of obese mice express higher levels of apelin mRNA. Increased apelin levels in mice also resulted in increased TNBC growth and metastases. Treatment with an apelin receptor (APLNR) antagonist, F13A, reduced TNBC growth and progression (Gourgue et al., 2020). Apelin-deficient BCa mice survive longer (Uribesalgo et al., 2019). It was also reported that inhibition of apelin reduces angiogenesis and, therefore, tumor growth. Apelin inhibition improves vessel function by reducing capillary leakage and tissue hypoxia and improving pericyte coverage (Uribesalgo et al., 2019). Furthermore, it was also reported that BCa cells under estrogen-deprived conditions express apelin-13 and its receptor APLNR and apelin-13 promotes cell growth while inhibiting autophagy. However, administration of exogenous estrogen reversed the effects. In addition, it was reported that apelin-13 deactivated the apoptotic kinase AMPK, indicating a novel pathway for estrogen-independent BCa growth, making it a potential therapeutic target (Bouchelaghem et al., 2022).

### 3.8 Lipocalin 2

Lipocalin 2 (LCN2), alternatively known as neutrophil gelatinase-associated lipocalin (NGAL), is a 25 kDa secretory peptide that belongs to the lipocalin family and is involved in the immunological response as well as the transportation of tiny hydrophobic compounds (Flower, 1996). It was recently reported that LCN2 is an adipokine secreted from human and mouse adipose tissues (Guo et al., 2010; Zhang et al., 2014). LCN2 expression levels have been found to be upregulated in BCa patients’ urine, serum, and tissue (Hu et al., 2018). Furthermore, several studies have demonstrated a correlation between LCN2 and BCa histological grade, disease relapse, metastasis, poor prognosis, and ER-negative status. In addition, compared to controls, patients with an invasive BCa diagnosis had higher serum levels of LCN2 (Bao et al., 2024). A study by Brian G. Drew et al. showed that fat-specific estrogen receptors alpha (ERα) deletion led to enhanced adipocyte size, fat pad weight, tissue expression, and circulating levels of released LCN2 in mice. Using luciferase reporter and chromatin immunoprecipitation, they reported that ERα interacts with the LCN2 promoter and suppresses its expression. Therefore, the study concluded that the reduction of ERα expression in adipose tissue induces obesity and is associated with increased adipocyte-specific LCN2 synthesis and higher LCN2 sensitivity in tumor cells, which in turn affects the course and severity of BCa (Drew et al., 2015). Furthermore, LCN2 expression was significantly higher in inflammatory BCa (IBC) tumors regardless of the molecular subtype than in non-inflammatory BCa tumors. In patients with inflammatory BCa, increased expression of LCN2 is associated with poor prognosis and a lower overall survival rate (Linjawi et al., 2018). The *in vivo* and *in vitro* studies by Yan et al. found that LCN2 is highly expressed in fat cells, which leads to insulin resistance. LCN2 serum levels are elevated in obese rodent models, and the reduction of LCN2 in 3T3-L1 adipocytes improves insulin action (Yan et al., 2007). These findings raise the possibility that LCN2 could be a useful noninvasive diagnostic and prognostic biomarker in BCa (Provatopoulou et al., 2009; Wenners et al., 2012; Christodoulatos et al., 2019).

*In vitro* and *in vivo* studies have shown that LCN2-tumorigenic and metastatic potential is induced via the elevation of EMT, cell migration, cell invasion, VEGF production, and angiogenesis (Yang et al., 2009; Ören et al., 2016; Christodoulatos et al., 2019). A notable upregulation of LCN2 expression has been observed in TNBC cell lines and various cell types of the TME, like endothelial cells, microvascular endothelial cells, stromal cells, lymphatic endothelial cells, fibroblasts, and M2-type macrophages. TNBC and stromal cell growth was markedly suppressed upon the addition of LCN2 antibody to the co-culture system (Malone et al., 2020). LCN2 has the ability to stimulate MMP-9 activity as the LCN2/MMP-9 complex can protect MMP-9 from protein degradation. MMP-9 localized levels may rise as a result of increased LCN2 expression in tumor cells or invading inflammatory cells (Yan et al., 2001). In BCa cells or mouse models, LCN2 silencing destabilizes the MMP-9/LCN2 complex and diminishes MMP-9 activity, cell invasion and migration, and VEGF production. It may also slow down the growth of tumors (Hu et al., 2018, p. 2). Upon phosphatase and tensin homolog (PTEN) knock down, MCF7 cells show increased expression of LCN2, upregulation of Snail, Twist, Slug, Zeb-1, Zeb-2, and P-cadherin, and downregulation of E-cadherin. This leads to the activation of EMT and metastatic progression (Chiang et al., 2016). Additionally, it was reported that in MDA-MB-231 cells, STAT3 stimulation increased LCN2 expression and triggered EMT dependent on the ERK pathway, promoting BCa lung metastasis (Tyagi et al., 2021). Expression of LCN2 proteins was suppressed by blocking the Wnt and NF-κB signaling pathways, indicating that these pathways are essential in LCN2-mediated tumor cell invasion and metastasis (Wang et al., 2011). In a study on BCa bone metastasis, osteoblasts and extracellular vesicles from BCa cells were co-cultured, and the results demonstrated a significant upregulation of LCN2 expression and stimulation of osteoclastogenesis (Loftus et al., 2020). LCN2 promoted IBC tumor aggressiveness *in vitro* and LCN2 depletion in IBC cell lines decreased colony formation, migration, and cancer stem cell populations. In mouse models of IBC, LCN2 depletion reduced tumor growth, skin invasion, and brain metastasis (Villodre et al., 2021).

## 4 Contributions of adipocytes to breast cancer pathogenesis and therapy

**FIGURE 1:**
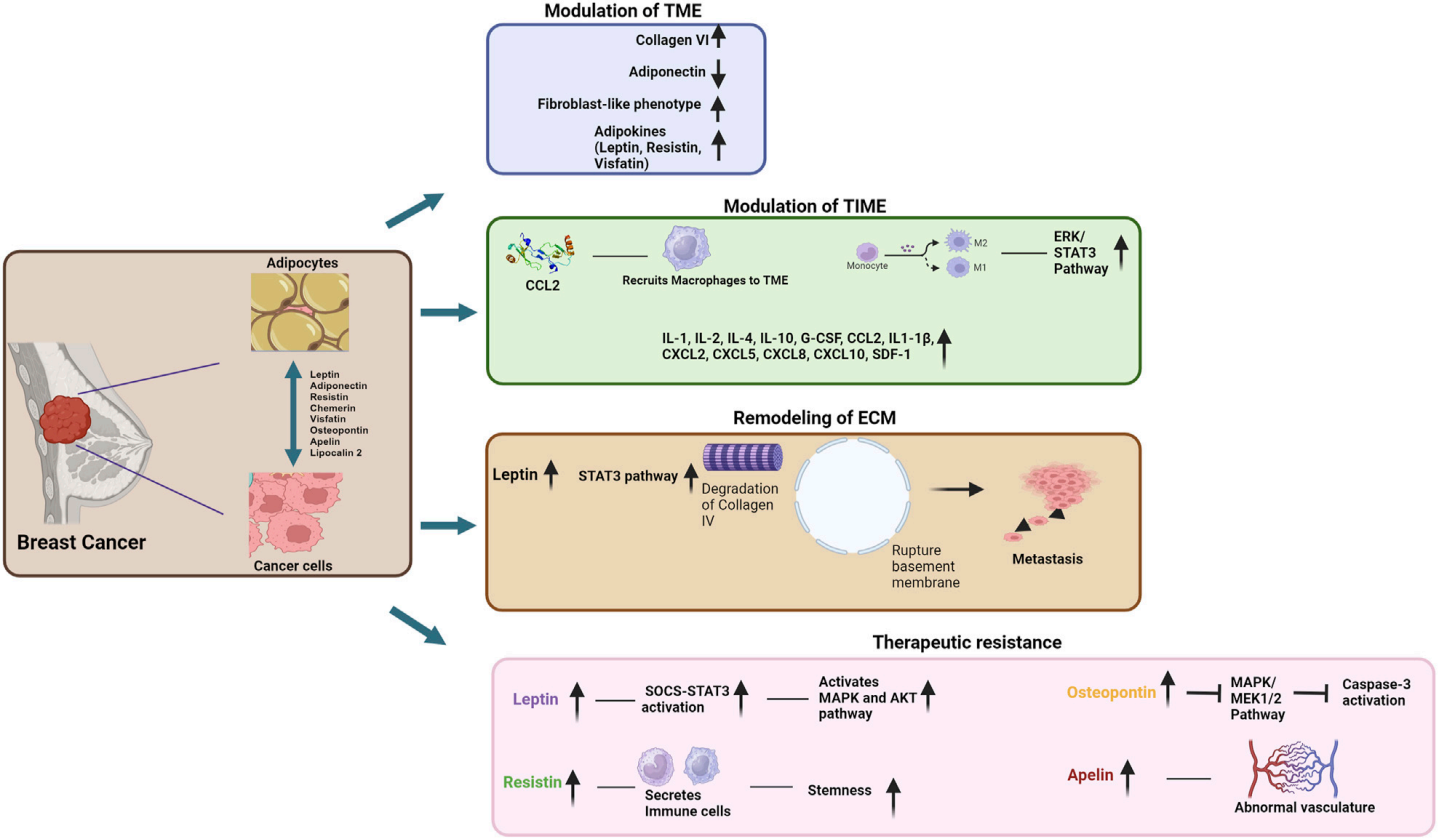
Schematic representation of the contributions of adipocytes to breast cancer pathogenesis and development of therapeutic resistance (Image created by BioRender).

Adipocytes within the TME promote BCa progression through various mechanisms. They play a crucial role in shaping the immune landscape within the tumor immune microenvironment (TIME) and remodeling of the extracellular matrix (ECM) within the TME, facilitating the invasion of tumor cells into the surrounding tissues and metastatic dissemination. They also aid in the development of therapeutic resistance. These multifaceted functions of the adipocytes contribute significantly to the pro-tumorigenic environment in BCa. In this section, we will discuss these pro-tumorigenic functions of the adipocytes in BCa TME. The effects of these functions on BCa growth and progression have been summarized in Figure 1.

### 4.1 Modulation of the tumor microenvironment

The crosstalk between adipocytes and cancer cells in the TME results in phenotypical and functional changes in both cell types, making obesity an independent risk factor for cancer progression (Wu et al., 2019). The TME is composed of mesenchymal support cells, immune cells, and matrix components. Adipocytes form the primary cellular component of the BCa microenvironment. Studies indicate that communication between adipocytes and cancer cells dictates the course of tumor progression (Dirat et al., 2011; Lapeire et al., 2014).

Compared to normal adipocytes, CAAs exhibit a distinct set of characteristics, including overexpression of collagen VI, low expression of adiponectin, fibroblast-like phenotypes, small and dispersed lipid droplets, and adipokines. Through the secretion of chemokine (C-C motif) ligand 2 (CCL2) (Fujisaki et al., 2015), chemokine (C-C motif) ligand 5 (CCL5, also known as RANTES) (Khalid et al., 2015; D’Esposito et al., 2016), VEGF, and leptin (De Palma et al., 2017), CAAs actively promote the invasion and metastasis of BCa cells. A study by D’Esposito delved into the mechanisms by which peritumoral human adipose tissue contributes to TNBC cell invasiveness and dissemination. The study demonstrated how co-culturing BCa cells with human adipocytes heightened the invasiveness of cancer cells through increased secretion of CCL5, suggesting that inhibiting CCL5 in the adipose microenvironment could be a promising strategy to curb highly malignant TNBC (D’Esposito et al., 2016). Moreover, adipocytes have emerged as key players in vascular remodeling and angiogenesis, contributing significantly to the regulation of angiogenesis through the secretion of angiogenic cytokines like VEGF and angiopoietin (Al-Ghadban et al., 2023).

### 4.2 Modulation of the tumor immune microenvironment

Myeloid-derived suppressor cells (MDSCs), tumor-associated macrophages (TAMs), natural killer cells (NKs), dendritic cells (DCs), and T cells are components of the tumor immune microenvironment (TIME) (Binnewies et al., 2018). Among the factors secreted by CAAs that may play a part in immune cell recruitment and functions are adiponectin, leptin, tumor necrosis factor alpha (TNFα), VEGF, plasminogen activator inhibitor-1 (PAI-1), secreted Frizzled Related Protein 5 (SFRP5), serum amyloid A (SAA), chemokine ligand 2 (CCL2), CCL5, IL-1β, IL-6, IL-8, and TNFα. (Larabee et al., 2020; Wu et al., 2021). Furthermore, because the TME is metabolically restrictive for immune cell infiltration, metabolic products derived from CAAs, such as lactate and fatty acids (FAs), also have a significant effect on the homeostasis and differentiation of immune cells, ultimately driving immune escape and tumor progression (Kedia-Mehta and Finlay, 2019). Neutrophils, the most prevalent and transient mediators of innate immunity, are a prime example of the significance of FAs in immunometabolism as they require FAs for correct differentiation and can take advantage of the interaction with CAAs (Riffelmacher et al., 2017). Apart from free FAs (FFAs), CAA may also stimulate the differentiation of neutrophils by raising their glycolytic flux and possibly enhancing the activity of tumor-associated neutrophils (TANs). It has been demonstrated that the recruitment of neutrophils in the TME by IL-8 derived from CAA is a step in the metastatic cascade in BCa (Vazquez Rodriguez et al., 2018).

Macrophages are a significant population in the TIME. Adipocytes affect adipose tissue macrophage polarization. Anti-inflammatory, immunosuppressive, and tumor-promoting M2 macrophage polarization was linked to FA uptake and oxidation (Rybinska et al., 2020). Furthermore, it has been demonstrated that adenosine and lactate released by CAAs can also increase M2 polarization by activating the ERK/STAT3 pathway (Mu et al., 2018; Wu et al., 2021). Resident M2 macrophages contribute to systemic metabolic homeostasis by playing an essential anti-inflammatory role in adipose tissue (Mantovani and Sica, 2010; Li et al., 2020). CCL2, also known as MCP-1 (monocyte chemoattractant protein-1), derived from adipocyte, recruits macrophages into the BCa TME, which increases the secretion of prostaglandin E2 (PGE2). PGE2 inhibits p53 expression and increases the expression of glucose transporters, glucose transporter 1 (GLUT1), glucose transporter 3 (GLUT3), and glucose transporter type 4 (GLUT4). This increases aerobic glycolysis and promotes the “Warburg effect” of BCa (Wu et al., 2019). Immune cells are also reported to be altered in adipose tissue. Under normal conditions, anti-inflammatory cytokine producing immune cells such as Tregs, T helper type 2 (TH2) cells, innate lymphoid type-2 cells (ILC2), and eosinophils are abundant. In contrast with obesity, the pro-inflammatory cytokine-producing T helper type 1 (TH1) cells, CD8^+^ cytotoxic T cells, and NK cells that produce IFNγ (Kammoun et al., 2014; Deng et al., 2016) become abundant in the adipose tissue due to excess leptin, which leads to M1 macrophage accumulation and polarization (Deng et al., 2016). A study by Núñez-Ruiz et al. reported a compromised anti-tumoral immune response in obesity with a significant decrease in infiltrating CD8^+^ T cells and M1/M2 macrophage ratio. In addition to a lower percentage of the number of intratumoral Tregs, the phenotypic analysis found an enrichment of CD39^+^, PD-1+, and CCR8+ cells compared with the draining lymph nodes. A significant rise in the percentage of peripheral Tregs supporting the immunosuppressive nature of the tumor was observed during tumor development in obese mice (Núñez-Ruiz et al., 2022).

Inflammation of adipose tissue is a hallmark of obesity, and the inflammatory TME affects cancer development at all stages, such as initiation, promotion, and progression. It starts with excessive energy intake, leading to energy imbalance and activation of various signaling pathways in metabolic tissues, including white adipose tissue (WAT). This results in the upregulation of inflammatory mediators and cytokines in the adipocyte tissue-resident cells (Gregor and Hotamisligil, 2011; Ellulu et al., 2017), contributing to a protumorigenic environment. Adipocytes secrete several interleukins, such as interleukin-1 (IL-1), IL-6, IL-8, and IL-10 influencing the immune response and inflammation. IL-1 plays a role in innate as well as adaptive immunity and mediates the inflammatory response in the presence of different stimuli (Fields et al., 2019). In BCa, IL-1 and IL-1β have been reported to induce tumorigenesis and bone metastasis by regulating the TME. In routine breast biopsy, IL-1 is not detected, but its level is significantly increased in BCa along with IL-2, IL-4, IL-10, and granulocyte colony stimulating factor (G-CSF) levels (Chavey et al., 2007). In addition, CAAs also secrete chemokines and other inflammatory cytokines, such as C-C motif chemokine ligand 2 (CCL2) and IL-1β, to stimulate adipocytes and promote cancer progression (Wu et al., 2021). Cancer cells also overexpress some pro-inflammatory factors, such as IL-6 and tumor necrosis factor α (TNF-α), that can stimulate adipocyte phenotype in a paracrine fashion (Rybinska et al., 2020). The coculturing of adipocytes with BCa cells resulted in an increased expression and secretion of IL-6 in adipocytes that promoted the invasion and migration of cancer cells (Lee et al., 2010; Chan et al., 2014; Kim et al., 2018). Also, the coculture of adipocytes with cancer cells led to an increased expression of pro-inflammatory factors, including IL-1β, and also showed an altered phenotype (Lapeire et al., 2014). IL-1β was reported to be a potential predictor of bone metastasis in BCa (Tulotta and Ottewell, 2018). In addition, the coculturing of adipocytes with cancer cells also increased TNF-α expression in adipocytes. The serum of BCa patients showed a significant elevation of TNF-α (Dirat et al., 2011). TNF-α participates in multiple cellular signaling pathways by binding to receptors, which correlates with inflammation and survival of BCa (Liu et al., 2016).

Adipocyte derived cytokines and chemokines include chemokine (C-X-C motif) ligand 2 (CXCL2), C-X-C motif chemokine 5 (CXCL5), C-X-C motif chemokine ligand 8 (CXCL8), C-X-C motif chemokine ligand 10 (CXCL10), stromal cell-derived factor 1 (SDF-1), monocyte chemoattractant protein-1 (MCP-1, CCL2), and macrophage inflammatory protein-1 Alpha (MIP-1α, CCL3). These are broadly studied in BCa and are usually associated with inflammation and tumorigenesis (Mulligan et al., 2013; Franzén et al., 2019; Romero-Moreno et al., 2019). The expression of MCP-1 is very high in BCa cells compared to normal epithelial cells of the breast (Mulligan et al., 2013; Franzén et al., 2019; Romero-Moreno et al., 2019). The chemokine secretion in BCa is responsible for increased invasiveness and metastasis (Lebel-Haziv et al., 2014). Excess nutrients in obesity result in a hypoxic and pro-inflammatory environment (Deng et al., 2016; Kolb and Zhang, 2020), leading to the polarization of M2 adipose tissue macrophages (ATM) to M1 type and the formation of “crown-like structures” (CLS), which is considered as a distinctive feature of adipose tissue inflammation. Increased CLS is evident in the breast adipose tissue in obese BCa patients. The CLS index-ratio in obese patients influences BCa recurrence and survival rates (Cildir et al., 2013; Faria et al., 2020) by influencing FAA release, NF-κB activation, and creation of a pro-inflammatory microenvironment (Jung and Choi, 2014). In addition, adipokines like visfatin, which is highly expressed in breast tumor tissues and in circulation, promote the differentiation of monocytic cells into TAMs that can indirectly influence BCa progression by enhancing the malignant behavior of BCa cells. The study by Wang et al. showed that in the BCa TME, monocytic cells, which include monocytes and macrophage precursors, can be recruited to the tumor site and differentiated into TAMs under the influence of visfatin. Once differentiated into TAMs, these cells can release cytokines such as CCL1, growth factors, and enzymes that support tumor growth and invasion (Wang et al., 2020; Wang et al., 2021). Like visfatin, leptin also plays a vital role in modulating the BCa TME. Leptin mediates the tumor-stromal cells’ crosstalk and plays a crucial role in the recruitment and sustenance of the photomural macrophages in the BCa TME (Gelsomino et al., 2020b). Pachynski et al. reported that overexpression and secretion of chemerin/RARRES2, commonly downregulated in BCa (Treeck et al., 2019), by tumor cells, significantly suppresses tumor growth *in vivo*. The study also reported an increase in NK and T cells in the chemerin-expressing tumor models compared to the control (Pachynski et al., 2019).

### 4.3 Remodeling of the extracellular matrix

The ECM is a three-dimensional intricate network of molecules and minerals that supports the structure and functions of cells and plays a crucial role in cell differentiation, migration, and survival. It forms a crucial aspect of the TME, and alterations in the ECM contribute significantly to BCa development (Bonnans et al., 2014).

During the development of BCa, the ECM undergoes numerous changes in composition and organization. Matrix proteins induced in the process include fibrillary collagens, fibronectin, specific laminins and proteoglycans, and matricellular proteins (Zhao et al., 2021). The ECM in cancer is defined by the content and proportion of different collagens that control tissue compliance, stiffness, porosity, viscoelasticity, and biochemical properties. The collagen-rich and laminin-rich basement membrane and stroma constitute the tumor cells’ boundary. The tumor cells can metastasize only if they penetrate the basement membrane (Cox, 2021). CAAs regulate the metastasis process by changing the content of multiple components in the ECM, such as collagen I, collagen IV, collagen VI, and fibronectin, by secreting many adipokines and cytokines, which influence the progression and metastasis of BCa (Queen et al., 2005; Dinca et al., 2021). CAAs secrete and process soluble ECM protein collagen VI, which activates NG2/chondroitin sulfate proteoglycan receptors on malignant ductal epithelial cells, activates AKT, β-catenin and cyclin 1, and promotes the remodeling of EMT and drives metastatic progression (Iyengar et al., 2005). The adipocytes also secrete leptin, which promotes the secretion of MMP-2 and MMP-9 by activating STAT3 signaling pathways, leading to the degradation of collagen IV, rupture of basement membrane and enabling metastatic dissemination of BCa cells (Juárez-Cruz et al., 2019). Co-culturing of adipocytes with BCa cells resulted in elevated levels of transforming growth factor-β (TGF-β) in BCa. TGF-β promotes CD73 expression and increases the adenosine content, decreasing collagen IV and increasing fibronectin in the ECM (Vasiukov et al., 2020). A study by Wang et al., 2019 reported that the CAAs derived exosomes could activate the hippo signaling pathways and therefore promote BCa progression in mice (Wang et al., 2019). These studies indicate that CAAs and CAA derived factors play important roles in ECM remodeling primarily by regulating the content and physiological status of different collagen and fibronectin molecules, leading to BCa progression and metastasis.

### 4.4 Promoting therapeutic resistance

Drug-resistant cancer cells pose significant clinical challenges. There are several ways by which obesity can affect the therapeutic outcomes in BCa. Dysfunctional adipocytes in obesity release an unbalanced mixture of adipokines (like insulin and leptin), metabolites (like cholesterol and free fatty acids), and cytokines (like TNFα and interleukins). Endocrine therapy is one of the most effective treatments for estrogen receptor-positive BCa. These adipokines can trigger resistance to endocrine therapy by triggering multiple signal transduction pathways and controlling genes linked to apoptosis (Chu et al., 2019). Obesity leads to increased estrogen production due to increased aromatase activity in adipose tissues (Goodwin, 2013). Aromatase Inhibitors (AI) primarily block the conversion of androgens to estrogen and are commonly used in treating hormone receptor-positive BCa. However, resistance to AIs can develop over time, leading to disease progression and poorer outcomes in BCa patients. It has been found that several adipokines and cytokines can reduce the efficacy of endocrine therapy *in vitro* by upregulating aromatase gene activity, which in turn can modifies estrogen synthesis (Chu et al., 2019; Barone et al., 2022). AI is less effective in obese patients because there is not enough suppression of serum estrogen, which leads to the development of drug resistance in these patients (Goodwin, 2013). Serum estrogen levels were reported to be much higher in obese patients (BMI> 35 kg/m^2^) both before and after taking AI medication compared to the non-obese subjects (BMI<25 kg/m^2^) (Folkerd et al., 2012; Pfeiler et al., 2013). Moreover, obesity causes a decrease in sex hormone-binding globulin, which inhibits estradiol and increases estrogen levels further (Cauley et al., 1989).

Additionally, obese individuals also have higher insulin levels, which causes BCa cells to produce more IGF-1. This, in turn, stimulates the RAS/RAF/MAPK and PI3K/AKT/mTOR signaling pathways, which leads to resistance to endocrine therapy (Surmacz and Burgaud, 1995; Campbell et al., 2001; Yee et al., 2020). Moreover, obesity is associated with an excess of reactive oxygen species (ROS) and proinflammatory molecules, which can promote tumor progression and cause resistance to hormone therapy (Xuan et al., 2014; Kastrati et al., 2020; Barone et al., 2022).

The increased expression of leptin is one of the reasons for therapeutic resistance in BCa. Leptin, via SOCS-STAT3 transcription activation, activates many downstream signaling pathways, such as MAPK and AKT pathways, resulting in increased angiogenesis, proliferation, decreased cancer cell death, and therapeutic resistance (Cirillo et al., 2008). It also activates estrogen receptors and human epidermal growth factor receptor 2, which promote tumor progression and resistance to targeted therapies (Fiorio et al., 2008; Soma et al., 2008). Gelsomino et al. reported that leptin contributes to AI-resistant BCa cell growth via activation of macrophages in the TME. The crosstalk between leptin signaling, development of resistance to AIs, and macrophage activation highlights the complexity of BCa progression and the importance of targeting multiple pathways for effective therapeutic outcomes. Strategies aimed at inhibiting leptin signaling, targeting aromatase activity, or modulating the TME may hold promise for overcoming AI resistance and improving outcomes for BCa patients (Gelsomino et al., 2020a). By increasing the BCa cell stemness, leptin also contributes to the development of drug resistance in BCa. Bougaret et al. showed that leptin, via STAT3, could upregulate the expression of carnitine palmitoyltransferase-1B (CPT1B) and the activity of fatty acid β-oxidation (FAO) in BCa stem cells (BCSCs), therefore increasing the stemness and drug resistance (Bougaret et al., 2018). Wang et al. showed that by blocking leptin, BCSCs could be resensitized to chemotherapy (Wang T. et al., 2018). Kim et al. found a strong relationship between leptin expression and poor outcomes for BCa patients receiving tamoxifen treatment. (Kim et al., 2006). The follow-up studies showed two significant mechanisms: (1) In BCa cell line, leptin causes the nuclear expression of estrogen receptor α (ERα), which interferes with the tamoxifen activity in MCF-7 cells; (2) Human epidermal growth factor receptor-2 (HER2) and the leptin/STAT3 pathway work together to regulate apoptosis-related genes, which causes BCa cells to become resistant to tamoxifen. (Chen et al., 2013). In another study, it was shown that the knockdown of long domain of the leptin receptor, which is encoded by the obese receptor b (ObRb) gene, significantly increases the inhibitory effect of tamoxifen on the survival and proliferation of BCa cells (Kim et al., 2006; Chen et al., 2013; Papanikolaou et al., 2015; Qian et al., 2015). In TNBC cells, leptin signaling weakens the effects of chemotherapy and elevates the expression of genes linked to chemoresistance and carcinogenesis (ABCB1, WNT4, ADHFE1, TBC1D3, LL22NC03, RDH5, and ITGB3). TNBCs were shown to become sensitive to chemotherapeutics upon OBR suppression. The survival and drug resistance of estrogen-receptor-negative breast cancer patients can, therefore, be predicted by looking at the co-expression of OBR and leptin-targeted genes. Targeting OBR signaling may increase the efficacy of chemotherapy (Lipsey et al., 2020). Also, the expression levels of the pre-B-cell leukemia homeobox transcription factor 3 (PBX3), a pioneer factor that governs divergent biological processes, were significantly upregulated in letrozole-resistant BCa cells and tissues. This upregulation was correlated with poor progression-free survival in patients. In a patient-derived xenograft model, the leptin-activated PBX3 expression in a STAT3-dependent manner. This indicates the novel role of leptin/PBX3 and their association with energy homeostasis and endocrine treatment therapy failure (i.e., letrozole resistance) in BCa (Pang et al., 2021).

There is scanty scientific evidence, however, about the relationship between adiponectin and drug efficacy or treatment resistance. A clinical study showed no association between treatment and adiponectin levels in BCa patients taking low-dose tamoxifen and fenretinide (Macis et al., 2012).

Resistin, secreted by immune cells such as monocytes, macrophages, and bone marrow cells, and is mainly found in the inflammatory regions, primarily increases the level of low-density lipoproteins (McTernan et al., 2002; Patel et al., 2003), induces BCa progression and elevates BCa cells stemness. The stemness of BCa cells was significantly impacted by resistin treatment, as evidenced by decreased CD24 cell surface expression, increased expression of CD44 and ALDH1, and enhanced cell mammosphere formation capabilities. Mechanistic investigations demonstrated that STAT3 activation mediated resistin-induced chemoresistance, apoptosis inhibition, and stemness in BCa cells. These findings reinforce resistin’s role in diverse clinical outcomes and offer further insight into its function in BCa biology (Deshmukh et al., 2017). It is also responsible for autophagy, leading to resistance to doxorubicin-induced apoptosis in BCa cells (Schwartz and Lazar, 2011).

The serum visfatin level (extracellular form) may predict a poor prognosis in BCa. The intracellular form of the enzyme is a rate-limiting enzyme in NAD + production (Assiri and Kamel, 2016). Resistance to anticancer agents and advancements in DNA repair progression are associated with elevated NAD + levels (Gujar et al., 2016).

In a large cohort of BCa patients receiving adjuvant chemotherapy, high OPN mRNA expression was linked to lower DFS and OS. OPN expression was linked to reduced therapeutic efficacy in BCa and could also be used to predict the efficiency of neoadjuvant chemotherapy in some patients (Gu and Zheng, 2017). OPN expression in BCa cells has been linked to the development of apoptosis resistance. In a study, Graessmann et al. have shown that OPN overexpressing mouse BCa cells are not only insensitive to apoptotic stimuli but also can confer apoptosis resistance in non OPN expressing BCa cells when cultured together by activating the MAPK/ERK signaling cascades and preventing caspase three degradation in these cells (Graessmann et al., 2007). Insua-Rodriguez et al. have shown how activation of JNK signaling in a subpopulation of BCa cells receiving chemotherapeutic agents induces OPN/SPP1 and Tenascin (TNC) to promote matrix remodeling, and therapeutic resistance in BCa (Insua-Rodríguez et al., 2018). OPN has also been directly linked to the development of drug resistance in BCa cells. OPN is a direct target of miR181 and suppresses p53-dependent transactivation and apoptosis in tumor cells, thereby regulating the sensitivity of MCF-7 cells to doxorubicin treatment (Han et al., 2019). In another study, Shevde et al. found that increased OPN expression is associated with increased tumor burden, enhanced vasculogenic mimicry, and worse prognosis in BCa patients. The study also identified hsa-mir-299-5p as a regulator of OPN expression in BCa (Shevde et al., 2010). The above findings indicate a strong role of OPN in metastasis and the development of drug resistance in BCa cells. More research is needed to develop an effective treatment strategy by manipulating OPN in the patients for BCa metastasis.

High expressions of intratumoral apelin have been shown to be associated with reduced response to neoadjuvant chemotherapy in BCa patients (Gourgue et al., 2021). Apelin has been primarily shown to be associated with angiogenesis in BCa as well as other cancers. Loss of apelin has been linked to angiogenic sprouting and tip cell formation. Azad et al. by single-cell RNA sequencing, have shown that apelin inhibition mainly prevents tip cell differentiation in experimental tumors in mice (Azad et al., 2022). Apelin is associated with the development of resistance to anti angiogenic therapy in BCa. Inhibition of apelin prevented the development of therapeutic resistance and antiangiogenic receptor tyrosine kinase (RTK) induced metastases of BCa cells (Uribesalgo et al., 2019). Gourgue et al., in their study involving 62 patients, however, concluded that apelin and obesity might be independently affecting the response to chemotherapy (Gourgue et al., 2021).

LCN2 expression was suppressed in drug-resistant 4T1 TNBC cells, and identified as a critical regulator of aggressive cell behavior in these cells. The study also identified increased bone morphogenetic protein (BMP) signaling and cross-talk between LCN2 and BMP as critical for determining drug sensitivity in these cells (Meurer et al., 2020).

The serine protease inhibitor, plasminogen activator inhibitor 1 (PA-1), plays a role in cell adhesion, migration, signal transduction, and anti-apoptosis (Gong et al., 2016). Adipocytes potentiate BCa metastasis by upregulating PAI-1 (Li et al., 2020). The high expression of PA-1 is associated with shorter disease-free survival in BCa (Witzel et al., 2014). In addition, the expression of PA-1 causes resistance to Src inhibitor via an increase in the secretion of CCL5 in HER2-positive BCa cells (Fang et al., 2018). The study by Su et al. reported that elevated SERPINE1 (PAI-1) expression and nuclear localization were observed in radioresistant BCa cells. It indicates that SERPINE1 might be a crucial factor for obesity associated radioresistance in TNBC (Su et al., 2023).

In addition, many growth factors are secreted by the adipose tissue, including VEGF, hepatocyte growth factor (HGF), nerve growth factor (NGF), insulin growth factor (IGF), and platelet derived growth factor (PDGF) (Peeraully et al., 2004; Pallua et al., 2009). The signaling mediated by these growth factors leads to the BCa progression, survival, angiogenesis, invasion, and metastasis (Witsch et al., 2010; Niepel et al., 2014). In addition, the crosstalk between these growth factors in BCa cells leads to resistance to therapies (Osborne and Schiff, 2003; Meyer et al., 2013; Voudouri et al., 2015; Linklater et al., 2016).

The breast adipose tissue matrix is enriched in collagen VI, which releases endotrophin. In BCa, endotrophin induces EMT. It was found that the endotrophin secreted by adipose tissue makes BCa cells resistant to cisplatin (Park et al., 2013). Another study reported that adipose-derived stem cells could promote the proliferation of chemotherapy residual TNBC cells by activating the ERK signaling cascade. The study further reported that adipocyte derived stem cells under the influence of the CXCR4/SDF-1α axis could migrate toward the chemo residual TNBC cells and promote their proliferation (Lyes et al., 2019). A study by Yeh et al. reported that ADSC secretes CXCL1 and promotes doxorubicin resistance in TNBC, which upregulates an ATP binding cassette transporter known as ATP-binding cassette superfamily G member 2 (ABCG2) (Yeh et al., 2017) and leads to the multidrug resistance (Calcagno et al., 2008). Adipocytes can also induce resistance to trastuzumab by interfering with interferon γ secretion by NK cells in HER2-expressing BCa cells (Duong et al., 2015). Adipocytes confer resistance to taxol in BCa cells by segmenting the autotaxin-lysophosphatidic acid signaling, which blocks the binding of taxol to tubulin (Samadi et al., 2011). Furthermore, adipose tissue adjacent to a growing tumor secretes IL-6, confers the resistance to radiotherapy, and upregulates checkpoint effector kinase (Chk1) responsible for resistance to radiation therapy (Bochet et al., 2011). Resistance to anti-VEGF treatments in obese BCa patients and mouse models could be due to the high levels of IL-6 and fibroblast growth factor-2 (FGF-2) (Incio et al., 2018).

## 5 Conclusion

Adipocytes play diverse roles in BCa progression. Understanding the intricate relationship between BCa, obesity, and weight loss is therefore crucial for devising prevention and treatment strategies. Even though the correlation between obesity and increased risk of developing BCa has been studied, the mechanisms behind this association is complex. The heterogeneity of cancer and adipose tissues, the dynamic nature of the adipose tissue, and the complex molecular signaling pathways involved in the adipocyte-mediated progression of BCa pose a significant challenge to our complete understanding of the process. Moreover, the impact of weight loss on BCa risk and treatment outcomes is still a new area of exploration. Reports suggest that there is an association between sustained weight loss and reduced BCa risk and better outcomes (Giarelli et al., 1986; Teras et al., 2020). Interestingly, results also show that restriction of calories and weight loss does not affect tumor progression (Demark-Wahnefried et al., 2020) or may lead to worse prognosis (Shang et al., 2021). Short-term pre-surgery weight loss interventions have shown mixed effects on tumor biology, and post-diagnosis weight loss has been associated with worse survival, though the reasons are not fully clear (Demark-Wahnefried et al., 2020; Puklin et al., 2023). Further investigation into the molecular pathways linking obesity to BCa development, and the effects of weight loss interventions on tumor progression and patient outcomes, is therefore warranted for informed targeted therapies and personalized healthcare approaches.

In this context, this review provides insight into the multifaceted role of the adipocytes in BCa progression and discusses the role of the various adipokines involved in the process. Further research should be conducted to identify the specific adipocyte-related targets and design therapeutic interventions tailored to the diverse molecular subtypes of BCa. Such endeavors will help expand our understanding of the role of obesity in BCa progression and increase the therapeutic options. One potential therapeutic approach involves inducing the browning of WAT. Converting white adipocytes to beige or brown adipocytes has been envisaged as a possible therapeutic strategy for cancers (Seki et al., 2022). This conversion also holds promise as a strategy for BCa, as it may promote energy consumption and expenditure and reduce the number of pro-inflammatory adipokines, creating a nonsupportive microenvironment for cancer cells. Therefore, understanding the molecular mechanisms underpinning the adipocyte-cancer cell interactions is needed to provide a foundation for developing targeted therapies that can promote this change.

## References

[B331] Al-Ghadban S. Walczak S. G. Isern S. U. Martin E. C. Herbst K. L. Bunnell B. A. (2023). Enhanced angiogenesis in HUVECs preconditioned with media from adipocytes differentiated from lipedema adipose stem cells *in vitro* . Int. J. Mol. Sci. 24, 13572. 10.3390/ijms241713572 37686378 PMC10487727

[B1] Andò S. Gelsomino L. Panza S. Giordano C. Bonofiglio D. Barone I. (2019). Obesity, leptin and breast cancer: epidemiological evidence and proposed mechanisms. Cancers (Basel) 11, 62. 10.3390/cancers11010062 30634494 PMC6356310

[B2] Annett S. Moore G. Robson T. (2020). Obesity and cancer metastasis: molecular and translational perspectives. Cancers (Basel) 12, 3798. 10.3390/cancers12123798 33339340 PMC7766668

[B3] Assiri A. M. A. Kamel H. F. M. (2016). Evaluation of diagnostic and predictive value of serum adipokines: leptin, resistin and visfatin in postmenopausal breast cancer. Obes. Res. Clin. Pract. 10, 442–453. 10.1016/j.orcp.2015.08.017 26388139

[B4] Assiri A. M. A. Kamel H. F. M. Hassanien M. F. R. (2015). Resistin, visfatin, adiponectin, and leptin: risk of breast cancer in pre- and postmenopausal saudi females and their possible diagnostic and predictive implications as novel biomarkers. Dis. Markers 2015, 253519. 10.1155/2015/253519 25838618 PMC4369904

[B5] Avtanski D. Garcia A. Caraballo B. Thangeswaran P. Marin S. Bianco J. (2019). Resistin induces breast cancer cells epithelial to mesenchymal transition (EMT) and stemness through both adenylyl cyclase-associated protein 1 (CAP1)-dependent and CAP1-independent mechanisms. Cytokine 120, 155–164. 10.1016/j.cyto.2019.04.016 31085453

[B6] Azad A. K. Campbell K. R. Zhabyeyev P. Oudit G. Y. Moore R. B. Murray A. G. (2022). Loss of apelin blocks the emergence of sprouting angiogenesis in experimental tumors. FASEB J. 36, e22560. 10.1096/fj.202200616RR 36165236

[B7] Bao Y. Yan Z. Shi N. Tian X. Li J. Li T. (2024). LCN2: versatile players in breast cancer. Biomed. Pharmacother. 171, 116091. 10.1016/j.biopha.2023.116091 38171248

[B8] Barone I. Caruso A. Gelsomino L. Giordano C. Bonofiglio D. Catalano S. (2022). Obesity and endocrine therapy resistance in breast cancer: mechanistic insights and perspectives. Obes. Rev. 23, e13358. 10.1111/obr.13358 34559450 PMC9285685

[B9] Behera R. Kumar V. Lohite K. Karnik S. Kundu G. C. (2010). Activation of JAK2/STAT3 signaling by osteopontin promotes tumor growth in human breast cancer cells. Carcinogenesis 31, 192–200. 10.1093/carcin/bgp289 19926637

[B10] Bergqvist M. Elebro K. Sandsveden M. Borgquist S. Rosendahl A. H. (2020). Effects of tumor-specific CAP1 expression and body constitution on clinical outcomes in patients with early breast cancer. Breast Cancer Res. 22, 67. 10.1186/s13058-020-01307-5 32560703 PMC7304201

[B11] Binnewies M. Roberts E. W. Kersten K. Chan V. Fearon D. F. Merad M. (2018). Understanding the tumor immune microenvironment (TIME) for effective therapy. Nat. Med. 24, 541–550. 10.1038/s41591-018-0014-x 29686425 PMC5998822

[B12] Bochet L. Meulle A. Imbert S. Salles B. Valet P. Muller C. (2011). Cancer-associated adipocytes promotes breast tumor radioresistance. Biochem. Biophys. Res. Commun. 411, 102–106. 10.1016/j.bbrc.2011.06.101 21712027

[B333] Bonnans C. Chou J. Werb Z. (2014). Remodelling the extracellular matrix in development and disease. Nat. Rev. Mol. Cell Biol. 15, 786–801. 10.1038/nrm3904 25415508 PMC4316204

[B13] Bouchelaghem R. Mader S. Gaboury L. Messarah M. Boumendjel M. Boumendjel A. (2022). Estrogens desensitize MCF-7 breast cancer cells to apelin-induced autophagy and enhanced growth under estrogen starvation: a possible implication in endocrine resistance. Cell Mol. Biol. (Noisy-le-grand) 68, 113–124. 10.14715/cmb/2022.68.9.18 36905266

[B14] Boucher J. Masri B. Daviaud D. Gesta S. Guigné C. Mazzucotelli A. (2005). Apelin, a newly identified adipokine up-regulated by insulin and obesity. Endocrinology 146, 1764–1771. 10.1210/en.2004-1427 15677759

[B15] Bougaret L. Delort L. Billard H. Le Huede C. Boby C. De la Foye A. (2018). Adipocyte/breast cancer cell crosstalk in obesity interferes with the anti-proliferative efficacy of tamoxifen. PLoS One 13, e0191571. 10.1371/journal.pone.0191571 29389973 PMC5794086

[B16] Bowers L. W. Rossi E. L. McDonell S. B. Doerstling S. S. Khatib S. A. Lineberger C. G. (2018). Leptin signaling mediates obesity-associated CSC enrichment and EMT in preclinical TNBC models. Mol. Cancer Res. 16, 869–879. 10.1158/1541-7786.MCR-17-0508 29453319 PMC5967653

[B17] Buonaiuto R. Napolitano F. Parola S. De Placido P. Forestieri V. Pecoraro G. (2022). Insight on the role of leptin: a bridge from obesity to breast cancer. Biomolecules 12, 1394. 10.3390/biom12101394 36291602 PMC9599120

[B18] Calcagno A. M. Fostel J. M. To K. K. W. Salcido C. D. Martin S. E. Chewning K. J. (2008). Single-step doxorubicin-selected cancer cells overexpress the ABCG2 drug transporter through epigenetic changes. Br. J. Cancer 98, 1515–1524. 10.1038/sj.bjc.6604334 18382425 PMC2386965

[B19] Campbell R. A. Bhat-Nakshatri P. Patel N. M. Constantinidou D. Ali S. Nakshatri H. (2001). Phosphatidylinositol 3-kinase/AKT-mediated activation of estrogen receptor alpha: a new model for anti-estrogen resistance. J. Biol. Chem. 276, 9817–9824. 10.1074/jbc.M010840200 11139588

[B20] Cauley J. A. Gutai J. P. Kuller L. H. LeDonne D. Powell J. G. (1989). The epidemiology of serum sex hormones in postmenopausal women. Am. J. Epidemiol. 129, 1120–1131. 10.1093/oxfordjournals.aje.a115234 2729251

[B21] Chan D. S. M. Vieira A. R. Aune D. Bandera E. V. Greenwood D. C. McTiernan A. (2014). Body mass index and survival in women with breast cancer-systematic literature review and meta-analysis of 82 follow-up studies. Ann. Oncol. 25, 1901–1914. 10.1093/annonc/mdu042 24769692 PMC4176449

[B22] Chavey C. Bibeau F. Gourgou-Bourgade S. Burlinchon S. Boissière F. Laune D. (2007). Oestrogen receptor negative breast cancers exhibit high cytokine content. Breast Cancer Res. 9, R15. 10.1186/bcr1648 17261184 PMC1851386

[B23] Chen G.-L. Luo Y. Eriksson D. Meng X. Qian C. Bäuerle T. (2016). High fat diet increases melanoma cell growth in the bone marrow by inducing osteopontin and interleukin 6. Oncotarget 7, 26653–26669. 10.18632/oncotarget.8474 27049717 PMC5042005

[B24] Chen X. Wang Y. (2011). Adiponectin and breast cancer. Med. Oncol. 28, 1288–1295. 10.1007/s12032-010-9617-x 20625941

[B25] Chen X. Zha X. Chen W. Zhu T. Qiu J. Røe O. D. (2013). Leptin attenuates the anti-estrogen effect of tamoxifen in breast cancer. Biomed. Pharmacother. 67, 22–30. 10.1016/j.biopha.2012.10.001 23199901

[B26] Chiang K.-C. Hsu S.-Y. Lin S.-J. Yeh C.-N. Pang J.-H. S. Wang S.-Y. (2016). PTEN insufficiency increases breast cancer cell metastasis *in vitro* and *in vivo* in a xenograft zebrafish model. Anticancer Res. 36, 3997–4005. 27466505

[B27] Choi H. M. Doss H. M. Kim K. S. (2020). Multifaceted physiological roles of adiponectin in inflammation and diseases. Int. J. Mol. Sci. 21, 1219. 10.3390/ijms21041219 32059381 PMC7072842

[B28] Choi J. Cha Y. J. Koo J. S. (2018). Adipocyte biology in breast cancer: from silent bystander to active facilitator. Prog. Lipid Res. 69, 11–20. 10.1016/j.plipres.2017.11.002 29175445

[B29] Christodoulatos G. S. Spyrou N. Kadillari J. Psallida S. Dalamaga M. (2019). The role of adipokines in breast cancer: current evidence and perspectives. Curr. Obes. Rep. 8, 413–433. 10.1007/s13679-019-00364-y 31637624

[B30] Chu D.-T. Phuong T. N. T. Tien N. L. B. Tran D.-K. Nguyen T.-T. Thanh V. V. (2019). The effects of adipocytes on the regulation of breast cancer in the tumor microenvironment: an update. Cells 8, 857. 10.3390/cells8080857 31398937 PMC6721665

[B31] Cildir G. Akıncılar S. C. Tergaonkar V. (2013). Chronic adipose tissue inflammation: all immune cells on the stage. Trends Mol. Med. 19, 487–500. 10.1016/j.molmed.2013.05.001 23746697

[B32] Cirillo D. Rachiglio A. M. la Montagna R. Giordano A. Normanno N. (2008). Leptin signaling in breast cancer: an overview. J. Cell Biochem. 105, 956–964. 10.1002/jcb.21911 18821585

[B33] Cleary M. P. Juneja S. C. Phillips F. C. Hu X. Grande J. P. Maihle N. J. (2004). Leptin receptor-deficient MMTV-TGF-alpha/Lepr(db)Lepr(db) female mice do not develop oncogene-induced mammary tumors. Exp. Biol. Med. (Maywood) 229, 182–193. 10.1177/153537020422900207 14734797

[B34] Cleary M. P. Phillips F. C. Getzin S. C. Jacobson T. L. Jacobson M. K. Christensen T. A. (2003). Genetically obese MMTV-TGF-alpha/Lep(ob)Lep(ob) female mice do not develop mammary tumors. Breast Cancer Res. Treat. 77, 205–215. 10.1023/a:1021891825399 12602920

[B35] Cleary M. P. Ray A. Rogozina O. P. Dogan S. Grossmann M. E. (2009). Targeting the adiponectin:leptin ratio for postmenopausal breast cancer prevention. Front. Biosci. Sch. Ed. 1, 329–357. 10.2741/S30 19482706

[B36] Cox T. R. (2021). The matrix in cancer. Nat. Rev. Cancer 21, 217–238. 10.1038/s41568-020-00329-7 33589810

[B37] Cui R.-R. Mao D.-A. Yi L. Wang C. Zhang X.-X. Xie H. (2010). Apelin suppresses apoptosis of human vascular smooth muscle cells via APJ/PI3-K/Akt signaling pathways. Amino Acids 39, 1193–1200. 10.1007/s00726-010-0555-x 20495838

[B38] Dakroub A. A Nasser S. Younis N. Bhagani H. Al-Dhaheri Y. Pintus G. (2020). Visfatin: a possible role in cardiovasculo-metabolic disorders. Cells 9, 2444. 10.3390/cells9112444 33182523 PMC7696687

[B39] Dalamaga M. Archondakis S. Sotiropoulos G. Karmaniolas K. Pelekanos N. Papadavid E. (2012). Could serum visfatin be a potential biomarker for postmenopausal breast cancer? Maturitas 71, 301–308. 10.1016/j.maturitas.2011.12.013 22261365

[B40] Dalamaga M. Karmaniolas K. Papadavid E. Pelekanos N. Sotiropoulos G. Lekka A. (2011). Elevated serum visfatin/nicotinamide phosphoribosyl-transferase levels are associated with risk of postmenopausal breast cancer independently from adiponectin, leptin, and anthropometric and metabolic parameters. Menopause 18, 1198–1204. 10.1097/gme.0b013e31821e21f5 21712732

[B41] Demark-Wahnefried W. Rogers L. Q. Gibson J. T. Harada S. Frugé A. D. Oster R. A. (2020). Randomized trial of weight loss in primary breast cancer: impact on body composition, circulating biomarkers and tumor characteristics. Int. J. Cancer 146, 2784–2796. 10.1002/ijc.32637 31442303 PMC7155016

[B332] De Palma M. Biziato D. Petrova T. V. (2017). Microenvironmental regulation of tumour angiogenesis. Nat. Rev. Cancer 17, 457–474. 10.1038/nrc.2017.51 28706266

[B42] Deng T. Lyon C. J. Bergin S. Caligiuri M. A. Hsueh W. A. (2016). Obesity, inflammation, and cancer. Annu. Rev. Pathol. 11, 421–449. 10.1146/annurev-pathol-012615-044359 27193454

[B43] Deshmukh S. K. Srivastava S. K. Bhardwaj A. Singh A. P. Tyagi N. Marimuthu S. (2015). Resistin and interleukin-6 exhibit racially-disparate expression in breast cancer patients, display molecular association and promote growth and aggressiveness of tumor cells through STAT3 activation. Oncotarget 6, 11231–11241. 10.18632/oncotarget.3591 25868978 PMC4484452

[B44] Deshmukh S. K. Srivastava S. K. Zubair H. Bhardwaj A. Tyagi N. Al-Ghadhban A. (2017). Resistin potentiates chemoresistance and stemness of breast cancer cells: implications for racially disparate therapeutic outcomes. Cancer Lett. 396, 21–29. 10.1016/j.canlet.2017.03.010 28302531 PMC5437742

[B330] D’Esposito V. Liguoro D. Ambrosio M. R. Collina F. Cantile M. Spinelli R. (2016). Adipose microenvironment promotes triple negative breast cancer cell invasiveness and dissemination by producing CCL5. Oncotarget 7, 24495–24509. 10.18632/oncotarget.8336 27027351 PMC5029717

[B45] Dinca S. C. Greiner D. Weidenfeld K. Bond L. Barkan D. Jorcyk C. L. (2021). Novel mechanism for OSM-promoted extracellular matrix remodeling in breast cancer: LOXL2 upregulation and subsequent ECM alignment. Breast Cancer Res. 23, 56. 10.1186/s13058-021-01430-x 34011405 PMC8132418

[B46] Dirat B. Bochet L. Dabek M. Daviaud D. Dauvillier S. Majed B. (2011). Cancer-associated adipocytes exhibit an activated phenotype and contribute to breast cancer invasion. Cancer Res. 71, 2455–2465. 10.1158/0008-5472.CAN-10-3323 21459803

[B47] Dogan S. Hu X. Zhang Y. Maihle N. J. Grande J. P. Cleary M. P. (2007). Effects of high-fat diet and/or body weight on mammary tumor leptin and apoptosis signaling pathways in MMTV-TGF-alpha mice. Breast Cancer Res. 9, R91. 10.1186/bcr1840 18162139 PMC2246166

[B48] Drew B. G. Hamidi H. Zhou Z. Villanueva C. J. Krum S. A. Calkin A. C. (2015). Estrogen receptor (ER)α-regulated lipocalin 2 expression in adipose tissue links obesity with breast cancer progression. J. Biol. Chem. 290, 5566–5581. 10.1074/jbc.M114.606459 25468909 PMC4342471

[B49] Duong M. N. Cleret A. Matera E.-L. Chettab K. Mathé D. Valsesia-Wittmann S. (2015). Adipose cells promote resistance of breast cancer cells to trastuzumab-mediated antibody-dependent cellular cytotoxicity. Breast Cancer Res. 17, 57. 10.1186/s13058-015-0569-0 25908175 PMC4482271

[B50] Elbaiomy M. A. Akl T. Elhelaly R. El-Beshbishi W. El Ghonemy M. S. Elzehery R. (2020). Osteopontin level and promoter polymorphism in patients with metastatic breast cancer. Curr. Oncol. 27, e444–e450. 10.3747/co.27.6449 33173383 PMC7606043

[B51] Ellulu M. S. Patimah I. Khaza’ai H. Rahmat A. Abed Y. (2017). Obesity and inflammation: the linking mechanism and the complications. Arch. Med. Sci. 13, 851–863. 10.5114/aoms.2016.58928 28721154 PMC5507106

[B52] El-Sagheer G. Gayyed M. Ahmad A. Abd El-Fattah A. Mohamed M. (2018). Expression of chemerin correlates with a poor prognosis in female breast cancer patients. Breast Cancer (Dove Med. Press) 10, 169–176. 10.2147/BCTT.S178181 30498371 PMC6207381

[B53] Fang H. Jin J. Huang D. Yang F. Guan X. (2018). PAI-1 induces Src inhibitor resistance via CCL5 in HER2-positive breast cancer cells. Cancer Sci. 109, 1949–1957. 10.1111/cas.13593 29601121 PMC5989873

[B54] Faria S. S. Corrêa L. H. Heyn G. S. de Sant’Ana L. P. Almeida R. das N. Magalhães K. G. (2020). Obesity and breast cancer: the role of crown-like structures in breast adipose tissue in tumor progression, prognosis, and therapy. J. Breast Cancer 23, 233–245. 10.4048/jbc.2020.23.e35 32595986 PMC7311368

[B55] Fields J. K. Günther S. Sundberg E. J. (2019). Structural basis of IL-1 family cytokine signaling. Front. Immunol. 10, 1412. 10.3389/fimmu.2019.01412 31281320 PMC6596353

[B56] Fiorio E. Mercanti A. Terrasi M. Micciolo R. Remo A. Auriemma A. (2008). Leptin/HER2 crosstalk in breast cancer: *in vitro* study and preliminary *in vivo* analysis. BMC Cancer 8, 305. 10.1186/1471-2407-8-305 18945363 PMC2588622

[B57] Flower D. R. (1996). The lipocalin protein family: structure and function. Biochem. J. 318 (Pt 1), 1–14. 10.1042/bj3180001 8761444 PMC1217580

[B58] Folkerd E. J. Dixon J. M. Renshaw L. A’Hern R. P. Dowsett M. (2012). Suppression of plasma estrogen levels by letrozole and anastrozole is related to body mass index in patients with breast cancer. J. Clin. Oncol. 30, 2977–2980. 10.1200/JCO.2012.42.0273 22802308

[B59] Franzén B. Alexeyenko A. Kamali-Moghaddam M. Hatschek T. Kanter L. Ramqvist T. (2019). Protein profiling of fine-needle aspirates reveals subtype-associated immune signatures and involvement of chemokines in breast cancer. Mol. Oncol. 13, 376–391. 10.1002/1878-0261.12410 30451357 PMC6360506

[B328] Fujisaki K. Fujimoto H. Sangai T. Nagashima T. Sakakibara M. Shiina N. (2015). Cancer-mediated adipose reversion promotes cancer cell migration via IL-6 and MCP-1. Breast Cancer Res. Treat. 150, 255–263. 10.1007/s10549-015-3318-2 25721605

[B60] Gelsomino L. Giordano C. Camera G. L. Sisci D. Marsico S. Campana A. (2020a). Leptin signaling contributes to aromatase inhibitor resistant breast cancer cell growth and activation of macrophages. Biomolecules 10, 543. 10.3390/biom10040543 32260113 PMC7226081

[B61] Gelsomino L. Naimo G. D. Malivindi R. Augimeri G. Panza S. Giordano C. (2020b). Knockdown of leptin receptor affects macrophage phenotype in the tumor microenvironment inhibiting breast cancer growth and progression. Cancers (Basel) 12, 2078. 10.3390/cancers12082078 32727138 PMC7464041

[B62] Georgiou G. P. Provatopoulou X. Kalogera E. Siasos G. Menenakos E. Zografos G. C. (2016). Serum resistin is inversely related to breast cancer risk in premenopausal women. Breast 29, 163–169. 10.1016/j.breast.2016.07.025 27521488

[B63] Ghaneialvar H. Shiri S. Kenarkoohi A. Fallah Vastani Z. Ahmadi A. Khorshidi A. (2022). Comparison of visfatin levels in patients with breast cancer and endometrial cancer with healthy individuals: a systematic review and meta-analysis. Health Sci. Rep. 5, e895. 10.1002/hsr2.895 36415563 PMC9674168

[B64] Gholinejad Z. Kheiripour N. Nourbakhsh M. Ilbeigi D. Behroozfar K. Hesari Z. (2017). Extracellular NAMPT/Visfatin induces proliferation through ERK1/2 and AKT and inhibits apoptosis in breast cancer cells. Peptides 92, 9–15. 10.1016/j.peptides.2017.04.007 28442350

[B65] Giarelli L. Stanta G. Delendi M. Sasco A. J. Ribolli E. (1986). Prevalence of female breast cancer observed in 517 unselected necropsies. Lancet 2, 864. 10.1016/s0140-6736(86)92901-6 2876309

[B66] Giordano C. Gelsomino L. Barone I. Panza S. Augimeri G. Bonofiglio D. (2019). Leptin modulates exosome biogenesis in breast cancer cells: an additional mechanism in cell-to-cell communication. J. Clin. Med. 8, 1027. 10.3390/jcm8071027 31336913 PMC6678227

[B67] Gong L. Proulle V. Fang C. Hong Z. Lin Z. Liu M. (2016). A specific plasminogen activator inhibitor-1 antagonist derived from inactivated urokinase. J. Cell Mol. Med. 20, 1851–1860. 10.1111/jcmm.12875 27197780 PMC4876229

[B68] Goodwin P. J. (2013). Obesity and endocrine therapy: host factors and breast cancer outcome. Breast 22 (Suppl. 2), S44–S47. 10.1016/j.breast.2013.07.008 24074791

[B69] Gourgue F. Derouane F. van Marcke C. Villar E. Dano H. Desmet L. (2021). Tumor apelin and obesity are associated with reduced neoadjuvant chemotherapy response in a cohort of breast cancer patients. Sci. Rep. 11, 9922. 10.1038/s41598-021-89385-z 33972642 PMC8110990

[B70] Gourgue F. Mignion L. Van Hul M. Dehaen N. Bastien E. Payen V. (2020). Obesity and triple-negative-breast-cancer: is apelin a new key target? J. Cell Mol. Med. 24, 10233–10244. 10.1111/jcmm.15639 32681609 PMC7520321

[B71] Graessmann M. Berg B. Fuchs B. Klein A. Graessmann A. (2007). Chemotherapy resistance of mouse WAP-SVT/t breast cancer cells is mediated by osteopontin, inhibiting apoptosis downstream of caspase-3. Oncogene 26, 2840–2850. 10.1038/sj.onc.1210096 17160024

[B72] Gregor M. F. Hotamisligil G. S. (2011). Inflammatory mechanisms in obesity. Annu. Rev. Immunol. 29, 415–445. 10.1146/annurev-immunol-031210-101322 21219177

[B73] Groeneveld M. P. Brierley G. V. Rocha N. M. Siddle K. Semple R. K. (2016). Acute knockdown of the insulin receptor or its substrates Irs1 and 2 in 3T3-L1 adipocytes suppresses adiponectin production. Sci. Rep. 6, 21105. 10.1038/srep21105 26888756 PMC4758029

[B74] Grossmann M. E. Cleary M. P. (2012). The balance between leptin and adiponectin in the control of carcinogenesis - focus on mammary tumorigenesis. Biochimie 94, 2164–2171. 10.1016/j.biochi.2012.06.013 22728769 PMC4296518

[B75] Gu L. Cao C. Fu J. Li Q. Li D.-H. Chen M.-Y. (2018). Serum adiponectin in breast cancer: a meta-analysis. Med. Baltim. 97, e11433. 10.1097/MD.0000000000011433 PMC6086546 30024516

[B76] Gu M. Zheng X. (2017). Osteopontin and vasculogenic mimicry formation are associated with response to neoadjuvant chemotherapy in advanced breast cancer. Onco Targets Ther. 10, 4121–4127. 10.2147/OTT.S129414 28860821 PMC5571838

[B77] Gujar A. D. Le S. Mao D. D. Dadey D. Y. A. Turski A. Sasaki Y. (2016). An NAD+-dependent transcriptional program governs self-renewal and radiation resistance in glioblastoma. Proc. Natl. Acad. Sci. U. S. A. 113, E8247–E8256. 10.1073/pnas.1610921114 27930300 PMC5187672

[B78] Guo H. Jin D. Zhang Y. Wright W. Bazuine M. Brockman D. A. (2010). Lipocalin-2 deficiency impairs thermogenesis and potentiates diet-induced insulin resistance in mice. Diabetes 59, 1376–1385. 10.2337/db09-1735 20332347 PMC2874698

[B79] Habata Y. Fujii R. Hosoya M. Fukusumi S. Kawamata Y. Hinuma S. (1999). Apelin, the natural ligand of the orphan receptor APJ, is abundantly secreted in the colostrum. Biochim. Biophys. Acta 1452, 25–35. 10.1016/s0167-4889(99)00114-7 10525157

[B80] Han B. Huang J. Han Y. Hao J. Wu X. Song H. (2019). The microRNA miR-181c enhances chemosensitivity and reduces chemoresistance in breast cancer cells via down-regulating osteopontin. Int. J. Biol. Macromol. 125, 544–556. 10.1016/j.ijbiomac.2018.12.075 30537505

[B81] Hao C. Wang Z. Gu Y. Jiang W. G. Cheng S. (2016). Prognostic value of osteopontin splice variant-c expression in breast cancers: a meta-analysis. Biomed. Res. Int. 2016, 7310694. 10.1155/2016/7310694 27462610 PMC4947640

[B82] Harborg S. Cronin-Fenton D. Jensen M.-B. R. Ahern T. P. Ewertz M. Borgquist S. (2023). Obesity and risk of recurrence in patients with breast cancer treated with aromatase inhibitors. JAMA Netw. Open 6, e2337780. 10.1001/jamanetworkopen.2023.37780 37831449 PMC10576219

[B83] Hu C. Yang K. Li M. Huang W. Zhang F. Wang H. (2018). Lipocalin 2: a potential therapeutic target for breast cancer metastasis. Onco Targets Ther. 11, 8099–8106. 10.2147/OTT.S181223 30519052 PMC6239117

[B84] Hu D. Cui Z. Peng W. Wang X. Chen Y. Wu X. (2022). Apelin is associated with clinicopathological parameters and prognosis in breast cancer patients. Arch. Gynecol. Obstet. 306, 1185–1195. 10.1007/s00404-022-06433-3 35249152

[B85] Hu G. Wang Z. Zhang R. Sun W. Chen X. (2021). The role of apelin/apelin receptor in energy metabolism and water homeostasis: a comprehensive narrative review. Front. Physiol. 12, 632886. 10.3389/fphys.2021.632886 33679444 PMC7928310

[B86] Huang J.-Y. Wang Y.-Y. Lo S. Tseng L.-M. Chen D.-R. Wu Y.-C. (2019). Visfatin mediates malignant behaviors through adipose-derived stem cells intermediary in breast cancer. Cancers (Basel) 12, 29. 10.3390/cancers12010029 31861872 PMC7016886

[B87] Huang Y. Jin Q. Su M. Ji F. Wang N. Zhong C. (2017). Leptin promotes the migration and invasion of breast cancer cells by upregulating ACAT2. Cell Oncol. (Dordr) 40, 537–547. 10.1007/s13402-017-0342-8 28770546 PMC13001567

[B88] Hung A. C. Lo S. Hou M.-F. Lee Y.-C. Tsai C.-H. Chen Y.-Y. (2016). Extracellular visfatin-promoted malignant behavior in breast cancer is mediated through c-abl and STAT3 activation. Clin. Cancer Res. 22, 4478–4490. 10.1158/1078-0432.CCR-15-2704 27036136

[B89] Icer M. A. Gezmen-Karadag M. (2018). The multiple functions and mechanisms of osteopontin. Clin. Biochem. 59, 17–24. 10.1016/j.clinbiochem.2018.07.003 30003880

[B90] Ilhan T. T. Kebapcilar A. Yilmaz S. A. Ilhan T. Kerimoglu O. S. Pekin A. T. (2015). Relations of serum visfatin and resistin levels with endometrial cancer and factors associated with its prognosis. Asian Pac J. Cancer Prev. 16, 4503–4508. 10.7314/apjcp.2015.16.11.4503 26107194

[B91] Incio J. Ligibel J. A. McManus D. T. Suboj P. Jung K. Kawaguchi K. (2018). Obesity promotes resistance to anti-VEGF therapy in breast cancer by up-regulating IL-6 and potentially FGF-2. Sci. Transl. Med. 10, eaag0945. 10.1126/scitranslmed.aag0945 29540614 PMC5936748

[B92] Insua-Rodríguez J. Pein M. Hongu T. Meier J. Descot A. Lowy C. M. (2018). Stress signaling in breast cancer cells induces matrix components that promote chemoresistant metastasis. EMBO Mol. Med. 10, e9003. 10.15252/emmm.201809003 30190333 PMC6180299

[B93] Iyengar P. Espina V. Williams T. W. Lin Y. Berry D. Jelicks L. A. (2005). Adipocyte-derived collagen VI affects early mammary tumor progression *in vivo*, demonstrating a critical interaction in the tumor/stroma microenvironment. J. Clin. Invest. 115, 1163–1176. 10.1172/JCI23424 15841211 PMC1077173

[B94] Jaberi S. A. Cohen A. D’Souza C. Abdulrazzaq Y. M. Ojha S. Bastaki S. (2021). Lipocalin-2: structure, function, distribution and role in metabolic disorders. Biomed. Pharmacother. 142, 112002. 10.1016/j.biopha.2021.112002 34463264

[B95] Jiralerspong S. Goodwin P. J. (2016). Obesity and breast cancer prognosis: evidence, challenges, and opportunities. J. Clin. Oncol. 34, 4203–4216. 10.1200/JCO.2016.68.4480 27903149

[B96] Juárez-Cruz J. C. Zuñiga-Eulogio M. D. Olea-Flores M. Castañeda-Saucedo E. Mendoza-Catalán M. Á. Ortuño-Pineda C. (2019). Leptin induces cell migration and invasion in a FAK-Src-dependent manner in breast cancer cells. Endocr. Connect. 8, 1539–1552. 10.1530/EC-19-0442 31671408 PMC6893313

[B97] Jung U. J. Choi M.-S. (2014). Obesity and its metabolic complications: the role of adipokines and the relationship between obesity, inflammation, insulin resistance, dyslipidemia and nonalcoholic fatty liver disease. Int. J. Mol. Sci. 15, 6184–6223. 10.3390/ijms15046184 24733068 PMC4013623

[B98] Kammoun H. L. Kraakman M. J. Febbraio M. A. (2014). Adipose tissue inflammation in glucose metabolism. Rev. Endocr. Metab. Disord. 15, 31–44. 10.1007/s11154-013-9274-4 24048715

[B99] Kastrati I. Joosten S. E. P. Semina S. E. Alejo L. H. Brovkovych S. D. Stender J. D. (2020). The NF-κB pathway promotes tamoxifen tolerance and disease recurrence in estrogen receptor-positive breast cancers. Mol. Cancer Res. 18, 1018–1027. 10.1158/1541-7786.MCR-19-1082 32245803 PMC7335344

[B100] Kaur J. Adya R. Tan B. K. Chen J. Randeva H. S. (2010). Identification of chemerin receptor (ChemR23) in human endothelial cells: chemerin-induced endothelial angiogenesis. Biochem. Biophys. Res. Commun. 391, 1762–1768. 10.1016/j.bbrc.2009.12.150 20044979

[B101] Kedia-Mehta N. Finlay D. K. (2019). Competition for nutrients and its role in controlling immune responses. Nat. Commun. 10, 2123. 10.1038/s41467-019-10015-4 31073180 PMC6509329

[B102] Kelesidis T. Kelesidis I. Chou S. Mantzoros C. S. (2010). Narrative review: the role of leptin in human physiology: emerging clinical applications. Ann. Intern Med. 152, 93–100. 10.7326/0003-4819-152-2-201001190-00008 20083828 PMC2829242

[B103] Kennedy A. J. Davenport A. P. (2018). International union of basic and clinical pharmacology CIII: chemerin receptors CMKLR1 (Chemerin1) and GPR1 (Chemerin2) nomenclature, pharmacology, and function. Pharmacol. Rev. 70, 174–196. 10.1124/pr.116.013177 29279348 PMC5744648

[B329] Khalid A. Wolfram J. Ferrari I. Mu C. Mai J. Yang Z. (2015). Recent Advances in Discovering the Role of CCL5 in Metastatic Breast Cancer. Mini. Rev. Med. Chem. 15, 1063–1072. 10.2174/138955751513150923094709 26420723 PMC4968951

[B104] Kim H. Lee J.-H. Lee S. K. Song N.-Y. Son S. H. Kim K. R. (2020). Chemerin treatment inhibits the growth and bone invasion of breast cancer cells. Int. J. Mol. Sci. 21, 2871. 10.3390/ijms21082871 32325994 PMC7216174

[B105] Kim H. S. Jung M. Choi S. K. Woo J. Piao Y. J. Hwang E. H. (2018). IL-6-mediated cross-talk between human preadipocytes and ductal carcinoma *in situ* in breast cancer progression. J. Exp. Clin. Cancer Res. 37, 200. 10.1186/s13046-018-0867-3 30134951 PMC6106749

[B106] Kim J. G. Kim E. O. Jeong B. R. Min Y. J. Park J. W. Kim E. S. (2010). Visfatin stimulates proliferation of MCF-7 human breast cancer cells. Mol. Cells 30, 341–345. 10.1007/s10059-010-0124-x 20848232

[B107] Kim Y. Kim S.-Y. Lee J. J. Seo J. Kim Y.-W. Koh S. H. (2006). Effects of the expression of leptin and leptin receptor (OBR) on the prognosis of early-stage breast cancers. Cancer Res. Treat. 38, 126–132. 10.4143/crt.2006.38.3.126 19771272 PMC2741678

[B108] Kolb R. Zhang W. (2020). Obesity and breast cancer: a case of inflamed adipose tissue. Cancers (Basel) 12, 1686. 10.3390/cancers12061686 32630445 PMC7352736

[B109] Kothari A. N. Arffa M. L. Chang V. Blackwell R. H. Syn W.-K. Zhang J. (2016). Osteopontin-A master regulator of epithelial-mesenchymal transition. J. Clin. Med. 5, 39. 10.3390/jcm5040039 27023622 PMC4850462

[B110] Kumar S. Patil H. S. Sharma P. Kumar D. Dasari S. Puranik V. G. (2012). Andrographolide inhibits osteopontin expression and breast tumor growth through down regulation of PI3 kinase/Akt signaling pathway. Curr. Mol. Med. 12, 952–966. 10.2174/156652412802480826 22804248

[B111] Lapeire L. Hendrix A. Lambein K. Van Bockstal M. Braems G. Van Den Broecke R. (2014). Cancer-associated adipose tissue promotes breast cancer progression by paracrine oncostatin M and Jak/STAT3 signaling. Cancer Res. 74, 6806–6819. 10.1158/0008-5472.CAN-14-0160 25252914

[B112] Larabee C. M. Neely O. C. Domingos A. I. (2020). Obesity: a neuroimmunometabolic perspective. Nat. Rev. Endocrinol. 16, 30–43. 10.1038/s41574-019-0283-6 31776456

[B113] Lebel-Haziv Y. Meshel T. Soria G. Yeheskel A. Mamon E. Ben-Baruch A. (2014). Breast cancer: coordinated regulation of CCL2 secretion by intracellular glycosaminoglycans and chemokine motifs. Neoplasia 16, 723–740. 10.1016/j.neo.2014.08.004 25246273 PMC4234876

[B114] Lee J. O. Kim N. Lee H. J. Lee Y. W. Kim S. J. Park S. H. (2016). Resistin, a fat-derived secretory factor, promotes metastasis of MDA-MB-231 human breast cancer cells through ERM activation. Sci. Rep. 6, 18923. 10.1038/srep18923 26729407 PMC4700449

[B115] Lee M.-J. Wu Y. Fried S. K. (2010). Adipose tissue remodeling in pathophysiology of obesity. Curr. Opin. Clin. Nutr. Metab. Care 13, 371–376. 10.1097/MCO.0b013e32833aabef 20531178 PMC3235038

[B116] Lee Y.-C. Chen Y.-J. Wu C.-C. Lo S. Hou M.-F. Yuan S.-S. F. (2012). Resistin expression in breast cancer tissue as a marker of prognosis and hormone therapy stratification. Gynecol. Oncol. 125, 742–750. 10.1016/j.ygyno.2012.02.032 22370603

[B117] Lee Y.-C. Yang Y.-H. Su J.-H. Chang H.-L. Hou M.-F. Yuan S.-S. F. (2011). High visfatin expression in breast cancer tissue is associated with poor survival. Cancer Epidemiol. Biomarkers Prev. 20, 1892–1901. 10.1158/1055-9965.EPI-11-0399 21784959

[B118] Li S.-J. Wei X.-H. Zhan X.-M. He J.-Y. Zeng Y.-Q. Tian X.-M. (2020). Adipocyte-derived leptin promotes PAI-1 -mediated breast cancer metastasis in a STAT3/miR-34a dependent manner. Cancers (Basel) 12, 3864. 10.3390/cancers12123864 33371368 PMC7767398

[B119] Linjawi S. AlGaithy Z. Sindi S. Hamdi N. Linjawi A. Alharbi M. (2018). Regulation of Lipocalin-2 oncogene and its impact on gene polymorphisms on breast cancer patients in Jeddah, Saudi Arabia. Saudi Med. J. 39, 558–563. 10.15537/smj.2018.6.22950 29915849 PMC6058746

[B120] Linklater E. S. Tovar E. A. Essenburg C. J. Turner L. Madaj Z. Winn M. E. (2016). Targeting MET and EGFR crosstalk signaling in triple-negative breast cancers. Oncotarget 7, 69903–69915. 10.18632/oncotarget.12065 27655711 PMC5342523

[B121] Lipsey C. C. Harbuzariu A. Robey R. W. Huff L. M. Gottesman M. M. Gonzalez-Perez R. R. (2020). Leptin signaling affects survival and chemoresistance of estrogen receptor negative breast cancer. Int. J. Mol. Sci. 21, 3794. 10.3390/ijms21113794 32471192 PMC7311967

[B122] Liu D. Wang X. Chen Z. (2016). Tumor necrosis factor-α, a regulator and therapeutic agent on breast cancer. Curr. Pharm. Biotechnol. 17, 486–494. 10.2174/1389201017666160301102713 26927216

[B123] Loftus A. Cappariello A. George C. Ucci A. Shefferd K. Green A. (2020). Extracellular vesicles from osteotropic breast cancer cells affect bone resident cells. J. Bone Min. Res. 35, 396–412. 10.1002/jbmr.3891 31610048

[B124] Lohmann A. E. Soldera S. V. Pimentel I. Ribnikar D. Ennis M. Amir E. (2021). Association of obesity with breast cancer outcome in relation to cancer subtypes: a meta-analysis. J. Natl. Cancer Inst. 113, 1465–1475. 10.1093/jnci/djab023 33620467 PMC8562970

[B125] Luhn P. Dallal C. M. Weiss J. M. Black A. Huang W.-Y. Lacey J. V. (2013). Circulating adipokine levels and endometrial cancer risk in the prostate, lung, colorectal, and ovarian cancer screening trial. Cancer Epidemiol. Biomarkers Prev. 22, 1304–1312. 10.1158/1055-9965.EPI-13-0258 23696194 PMC3819202

[B126] Lund S. A. Giachelli C. M. Scatena M. (2009). The role of osteopontin in inflammatory processes. J. Cell Commun. Signal 3, 311–322. 10.1007/s12079-009-0068-0 19798593 PMC2778587

[B127] Lyes M. A. Payne S. Ferrell P. Pizzo S. V. Hollenbeck S. T. Bachelder R. E. (2019). Adipose stem cell crosstalk with chemo-residual breast cancer cells: implications for tumor recurrence. Breast Cancer Res. Treat. 174, 413–422. 10.1007/s10549-018-05103-w 30594967 PMC6422973

[B128] Macis D. Gandini S. Guerrieri-Gonzaga A. Johansson H. Magni P. Ruscica M. (2012). Prognostic effect of circulating adiponectin in a randomized 2 x 2 trial of low-dose tamoxifen and fenretinide in premenopausal women at risk for breast cancer. J. Clin. Oncol. 30, 151–157. 10.1200/JCO.2011.35.2237 22162577 PMC3255561

[B129] Malone M. K. Smrekar K. Park S. Blakely B. Walter A. Nasta N. (2020). Cytokines secreted by stromal cells in TNBC microenvironment as potential targets for cancer therapy. Cancer Biol. Ther. 21, 560–569. 10.1080/15384047.2020.1739484 32213106 PMC7515526

[B130] Mantovani A. Sica A. (2010). Macrophages, innate immunity and cancer: balance, tolerance, and diversity. Curr. Opin. Immunol. 22, 231–237. 10.1016/j.coi.2010.01.009 20144856

[B131] Martinetti A. Bajetta E. Ferrari L. Zilembo N. Seregni E. Del Vecchio M. (2004). Osteoprotegerin and osteopontin serum values in postmenopausal advanced breast cancer patients treated with anastrozole. Endocr. Relat. Cancer 11, 771–779. 10.1677/erc.1.00775 15613451

[B132] Maurizi G. Babini L. Della Guardia L. (2018). Potential role of microRNAs in the regulation of adipocytes liposecretion and adipose tissue physiology. J. Cell Physiol. 233, 9077–9086. 10.1002/jcp.26523 29932216

[B133] Mauro L. Pellegrino M. De Amicis F. Ricchio E. Giordano F. Rizza P. (2014). Evidences that estrogen receptor α interferes with adiponectin effects on breast cancer cell growth. Cell Cycle 13, 553–564. 10.4161/cc.27455 24335340

[B134] McTernan C. L. McTernan P. G. Harte A. L. Levick P. L. Barnett A. H. Kumar S. (2002). Resistin, central obesity, and type 2 diabetes. Lancet 359, 46–47. 10.1016/s0140-6736(02)07281-1 11809189

[B135] Meurer S. K. Tezcan O. Lammers T. Weiskirchen R. (2020). Differential regulation of Lipocalin 2 (LCN2) in doxorubicin-resistant 4T1 triple negative breast cancer cells. Cell Signal 74, 109731. 10.1016/j.cellsig.2020.109731 32758668

[B136] Meyer A. S. Miller M. A. Gertler F. B. Lauffenburger D. A. (2013). The receptor AXL diversifies EGFR signaling and limits the response to EGFR-targeted inhibitors in triple-negative breast cancer cells. Sci. Signal 6, ra66. 10.1126/scisignal.2004155 23921085 PMC3947921

[B137] Moseti D. Regassa A. Kim W.-K. (2016). Molecular regulation of adipogenesis and potential anti-adipogenic bioactive molecules. Int. J. Mol. Sci. 17, 124. 10.3390/ijms17010124 26797605 PMC4730365

[B138] Mu X. Shi W. Xu Y. Xu C. Zhao T. Geng B. (2018). Tumor-derived lactate induces M2 macrophage polarization via the activation of the ERK/STAT3 signaling pathway in breast cancer. Cell Cycle 17, 428–438. 10.1080/15384101.2018.1444305 29468929 PMC5927648

[B139] Mulligan A. M. Raitman I. Feeley L. Pinnaduwage D. Nguyen L. T. O’Malley F. P. (2013). Tumoral lymphocytic infiltration and expression of the chemokine CXCL10 in breast cancers from the Ontario Familial Breast Cancer Registry. Clin. Cancer Res. 19, 336–346. 10.1158/1078-0432.CCR-11-3314 23213058 PMC3548938

[B140] Naimo G. D. Paolì A. Giordano F. Forestiero M. Panno M. L. Andò S. (2023). Unraveling the role of adiponectin receptors in obesity-related breast cancer. Int. J. Mol. Sci. 24, 8907. 10.3390/ijms24108907 37240258 PMC10218957

[B141] Nehme R. Diab-Assaf M. Decombat C. Delort L. Caldefie-Chezet F. (2022). Targeting adiponectin in breast cancer. Biomedicines 10, 2958. 10.3390/biomedicines10112958 36428526 PMC9687473

[B142] Nepal S. Kim M. J. Hong J. T. Kim S. H. Sohn D.-H. Lee S. H. (2015). Autophagy induction by leptin contributes to suppression of apoptosis in cancer cells and xenograft model: involvement of p53/FoxO3A axis. Oncotarget 6, 7166–7181. 10.18632/oncotarget.3347 25704884 PMC4466676

[B143] Nieman K. M. Kenny H. A. Penicka C. V. Ladanyi A. Buell-Gutbrod R. Zillhardt M. R. (2011). Adipocytes promote ovarian cancer metastasis and provide energy for rapid tumor growth. Nat. Med. 17, 1498–1503. 10.1038/nm.2492 22037646 PMC4157349

[B144] Niepel M. Hafner M. Pace E. A. Chung M. Chai D. H. Zhou L. (2014). Analysis of growth factor signaling in genetically diverse breast cancer lines. BMC Biol. 12, 20. 10.1186/1741-7007-12-20 24655548 PMC4234128

[B145] Niu J. Jiang L. Guo W. Shao L. Liu Y. Wang L. (2013). The association between leptin level and breast cancer: a meta-analysis. PLoS One 8, e67349. 10.1371/journal.pone.0067349 23826274 PMC3694967

[B146] Núñez-Ruiz A. Sánchez-Brena F. López-Pacheco C. Acevedo-Domínguez N. A. Soldevila G. (2022). Obesity modulates the immune macroenvironment associated with breast cancer development. PLoS One 17, e0266827. 10.1371/journal.pone.0266827 35472214 PMC9041840

[B147] Ören B. Urosevic J. Mertens C. Mora J. Guiu M. Gomis R. R. (2016). Tumour stroma-derived lipocalin-2 promotes breast cancer metastasis. J. Pathol. 239, 274–285. 10.1002/path.4724 27038000

[B148] Osborne C. K. Schiff R. (2003). Growth factor receptor cross-talk with estrogen receptor as a mechanism for tamoxifen resistance in breast cancer. Breast 12, 362–367. 10.1016/s0960-9776(03)00137-1 14659106

[B149] Ouchi N. Walsh K. (2007). Adiponectin as an anti-inflammatory factor. Clin. Chim. Acta 380, 24–30. 10.1016/j.cca.2007.01.026 17343838 PMC2755046

[B150] Pachynski R. K. Wang P. Salazar N. Zheng Y. Nease L. Rosalez J. (2019). Chemerin suppresses breast cancer growth by recruiting immune effector cells into the tumor microenvironment. Front. Immunol. 10, 983. 10.3389/fimmu.2019.00983 31139180 PMC6518384

[B151] Pallua N. Pulsfort A. K. Suschek C. Wolter T. P. (2009). Content of the growth factors bFGF, IGF-1, VEGF, and PDGF-BB in freshly harvested lipoaspirate after centrifugation and incubation. Plast. Reconstr. Surg. 123, 826–833. 10.1097/PRS.0b013e318199ef31 19319045

[B152] Pan H. Deng L.-L. Cui J.-Q. Shi L. Yang Y.-C. Luo J.-H. (2018). Association between serum leptin levels and breast cancer risk: an updated systematic review and meta-analysis. Med. Baltim. 97, e11345. 10.1097/MD.0000000000011345 PMC6076146 29979411

[B153] Pang Z.-Y. Wei Y.-T. Shang M.-Y. Li S. Li Y. Jin Q.-X. (2021). Leptin-elicited PBX3 confers letrozole resistance in breast cancer. Endocr. Relat. Cancer 28, 173–189. ERC-20-0328.R2. 10.1530/ERC-20-0328 33608482

[B154] Papakonstantinou E. Piperigkou Z. Karamanos N. K. Zolota V. (2022). Altered adipokine expression in tumor microenvironment promotes development of triple negative breast cancer. Cancers (Basel) 14, 4139. 10.3390/cancers14174139 36077676 PMC9454958

[B155] Papanikolaou V. Stefanou N. Dubos S. Papathanasiou I. Palianopoulou M. Valiakou V. (2015). Synergy of leptin/STAT3 with HER2 receptor induces tamoxifen resistance in breast cancer cells through regulation of apoptosis-related genes. Cell Oncol. (Dordr) 38, 155–164. 10.1007/s13402-014-0213-5 25539992 PMC13004254

[B156] Parida S. Siddharth S. Sharma D. (2019). Adiponectin, obesity, and cancer: clash of the bigwigs in health and disease. Int. J. Mol. Sci. 20, 2519. 10.3390/ijms20102519 31121868 PMC6566909

[B157] Park H.-J. Kim S.-R. Kim S. S. Wee H.-J. Bae M.-K. Ryu M. H. (2014). Visfatin promotes cell and tumor growth by upregulating Notch1 in breast cancer. Oncotarget 5, 5087–5099. 10.18632/oncotarget.2086 24970818 PMC4148124

[B158] Park J. Morley T. S. Scherer P. E. (2013). Inhibition of endotrophin, a cleavage product of collagen VI, confers cisplatin sensitivity to tumours. EMBO Mol. Med. 5, 935–948. 10.1002/emmm.201202006 23629957 PMC3779453

[B159] Park J. Scherer P. E. (2011). Leptin and cancer: from cancer stem cells to metastasis. Endocr. Relat. Cancer 18, C25–C29. 10.1530/ERC-11-0163 21680729 PMC4110513

[B160] Park J. W. Zhao L. Willingham M. C. Cheng S.-Y. (2017). Inhibition of STAT3 signaling blocks obesity-induced mammary hyperplasia in a mouse model. Am. J. Cancer Res. 7, 727–739. 28401024 PMC5385655

[B161] Park S. J. Yu Y. Zides C. G. Beyak M. J. (2022). Mechanisms of reduced leptin-mediated satiety signaling during obesity. Int. J. Obes. (Lond) 46, 1212–1221. 10.1038/s41366-022-01079-2 35241786

[B162] Patel L. Buckels A. C. Kinghorn I. J. Murdock P. R. Holbrook J. D. Plumpton C. (2003). Resistin is expressed in human macrophages and directly regulated by PPAR gamma activators. Biochem. Biophys. Res. Commun. 300, 472–476. 10.1016/s0006-291x(02)02841-3 12504108

[B163] Peeraully M. R. Jenkins J. R. Trayhurn P. (2004). NGF gene expression and secretion in white adipose tissue: regulation in 3T3-L1 adipocytes by hormones and inflammatory cytokines. Am. J. Physiol. Endocrinol. Metab. 287, E331–E339. 10.1152/ajpendo.00076.2004 15100092

[B164] Pfeiler G. Königsberg R. Hadji P. Fitzal F. Maroske M. Dressel-Ban G. (2013). Impact of body mass index on estradiol depletion by aromatase inhibitors in postmenopausal women with early breast cancer. Br. J. Cancer 109, 1522–1527. 10.1038/bjc.2013.499 24002592 PMC3777005

[B165] Provatopoulou X. Gounaris A. Kalogera E. Zagouri F. Flessas I. Goussetis E. (2009). Circulating levels of matrix metalloproteinase-9 (MMP-9), neutrophil gelatinase-associated lipocalin (NGAL) and their complex MMP-9/NGAL in breast cancer disease. BMC Cancer 9, 390. 10.1186/1471-2407-9-390 19889214 PMC2775750

[B166] Psyrri A. Kalogeras K. T. Wirtz R. M. Kouvatseas G. Karayannopoulou G. Goussia A. (2017). Association of osteopontin with specific prognostic factors and survival in adjuvant breast cancer trials of the Hellenic Cooperative Oncology Group. J. Transl. Med. 15, 30. 10.1186/s12967-017-1134-7 28193231 PMC5304396

[B167] Puklin L. S. Li F. Cartmel B. Zhao J. Sanft T. Lisevick A. (2023). Post-diagnosis weight trajectories and mortality among women with breast cancer. NPJ Breast Cancer 9, 98. 10.1038/s41523-023-00603-5 38042922 PMC10693588

[B168] Qian Y. Shi D. Qiu J. Zhu F. Qian J. He S. (2015). ObRb downregulation increases breast cancer cell sensitivity to tamoxifen. Tumour Biol. 36, 6813–6821. 10.1007/s13277-015-3375-5 25846733

[B169] Queen M. M. Ryan R. E. Holzer R. G. Keller-Peck C. R. Jorcyk C. L. (2005). Breast cancer cells stimulate neutrophils to produce oncostatin M: potential implications for tumor progression. Cancer Res. 65, 8896–8904. 10.1158/0008-5472.CAN-05-1734 16204061

[B170] Raja R. Kale S. Thorat D. Soundararajan G. Lohite K. Mane A. (2014). Hypoxia-driven osteopontin contributes to breast tumor growth through modulation of HIF1α-mediated VEGF-dependent angiogenesis. Oncogene 33, 2053–2064. 10.1038/onc.2013.171 23728336

[B171] Rajput P. K. Varghese J. F. Srivastava A. K. Kumar U. Yadav U. C. S. (2023). Visfatin-induced upregulation of lipogenesis via EGFR/AKT/GSK3β pathway promotes breast cancer cell growth. Cell Signal 107, 110686. 10.1016/j.cellsig.2023.110686 37084841

[B172] Riffelmacher T. Clarke A. Richter F. C. Stranks A. Pandey S. Danielli S. (2017). Autophagy-dependent generation of free fatty acids is critical for normal neutrophil differentiation. Immunity 47, 466–480. 10.1016/j.immuni.2017.08.005 28916263 PMC5610174

[B173] Romero-Moreno R. Curtis K. J. Coughlin T. R. Miranda-Vergara M. C. Dutta S. Natarajan A. (2019). The CXCL5/CXCR2 axis is sufficient to promote breast cancer colonization during bone metastasis. Nat. Commun. 10, 4404. 10.1038/s41467-019-12108-6 31562303 PMC6765048

[B174] Rybinska I. Agresti R. Trapani A. Tagliabue E. Triulzi T. (2020). Adipocytes in breast cancer, the thick and the thin. Cells 9, 560. 10.3390/cells9030560 32120856 PMC7140407

[B175] Rybinska I. Mangano N. Tagliabue E. Triulzi T. (2021). Cancer-associated adipocytes in breast cancer: causes and consequences. Int. J. Mol. Sci. 22, 3775. 10.3390/ijms22073775 33917351 PMC8038661

[B176] Samadi N. Bekele R. T. Goping I. S. Schang L. M. Brindley D. N. (2011). Lysophosphatidate induces chemo-resistance by releasing breast cancer cells from taxol-induced mitotic arrest. PLoS One 6, e20608. 10.1371/journal.pone.0020608 21647386 PMC3103588

[B177] Sanchez-Infantes D. White U. A. Elks C. M. Morrison R. F. Gimble J. M. Considine R. V. (2014). Oncostatin m is produced in adipose tissue and is regulated in conditions of obesity and type 2 diabetes. J. Clin. Endocrinol. Metab. 99, E217–E225. 10.1210/jc.2013-3555 24297795 PMC3913819

[B178] Sánchez-Jiménez F. Pérez-Pérez A. de la Cruz-Merino L. Sánchez-Margalet V. (2019). Obesity and breast cancer: role of leptin. Front. Oncol. 9, 596. 10.3389/fonc.2019.00596 31380268 PMC6657346

[B179] Sato Y. Sakurai M. Noda E. Tanabe O. Hojo M. Kitamura A. (1991). A case of small cell carcinoma originating at the site of pneumoplication of giant bulla five years later. Kyobu Geka 44, 587–589. 1653376

[B180] Saxena N. K. Sharma D. (2013). Multifaceted leptin network: the molecular connection between obesity and breast cancer. J. Mammary Gland. Biol. Neoplasia 18, 309–320. 10.1007/s10911-013-9308-2 24214584 PMC4747028

[B181] Saxena N. K. Vertino P. M. Anania F. A. Sharma D. (2007). leptin-induced growth stimulation of breast cancer cells involves recruitment of histone acetyltransferases and mediator complex to CYCLIN D1 promoter via activation of Stat3. J. Biol. Chem. 282, 13316–13325. 10.1074/jbc.M609798200 17344214 PMC2923657

[B182] Schwartz D. R. Lazar M. A. (2011). Human resistin: found in translation from mouse to man. Trends Endocrinol. Metab. 22, 259–265. 10.1016/j.tem.2011.03.005 21497511 PMC3130099

[B183] Seki T. Yang Y. Sun X. Lim S. Xie S. Guo Z. (2022). Brown-fat-mediated tumour suppression by cold-altered global metabolism. Nature 608, 421–428. 10.1038/s41586-022-05030-3 35922508 PMC9365697

[B184] Shang L. Hattori M. Fleming G. Jaskowiak N. Hedeker D. Olopade O. I. (2021). Impact of post-diagnosis weight change on survival outcomes in Black and White breast cancer patients. Breast Cancer Res. 23, 18. 10.1186/s13058-021-01397-9 33541403 PMC7863526

[B185] Shevde L. A. Das S. Clark D. W. Samant R. S. (2010). Osteopontin: an effector and an effect of tumor metastasis. Curr. Mol. Med. 10, 71–81. 10.2174/156652410791065381 20205680 PMC6869338

[B186] Siegel R. L. Giaquinto A. N. Jemal A. (2024). Cancer statistics, 2024. CA Cancer J. Clin. 74, 12–49. 10.3322/caac.21820 38230766

[B187] Siegel R. L. Miller K. D. Jemal A. (2020). Cancer statistics, 2020. CA Cancer J. Clin. 70, 7–30. 10.3322/caac.21590 31912902

[B188] Soma D. Kitayama J. Yamashita H. Miyato H. Ishikawa M. Nagawa H. (2008). Leptin augments proliferation of breast cancer cells via transactivation of HER2. J. Surg. Res. 149, 9–14. 10.1016/j.jss.2007.10.012 18262553

[B189] Song Y. Zhu X. Lin Z. Luo L. Wen D. (2021). The potential value of serum chemerin in patients with breast cancer. Sci. Rep. 11, 6564. 10.1038/s41598-021-85986-w 33753802 PMC7985153

[B190] Spyrou N. Avgerinos K. I. Mantzoros C. S. Dalamaga M. (2018). Classic and novel adipocytokines at the intersection of obesity and cancer: diagnostic and therapeutic strategies. Curr. Obes. Rep. 7, 260–275. 10.1007/s13679-018-0318-7 30145771

[B191] Steppan C. M. Bailey S. T. Bhat S. Brown E. J. Banerjee R. R. Wright C. M. (2001). The hormone resistin links obesity to diabetes. Nature 409, 307–312. 10.1038/35053000 11201732

[B192] Su Y.-H. Wu Y.-Z. Ann D. K. Chen J. L.-Y. Kuo C.-Y. (2023). Obesity promotes radioresistance through SERPINE1-mediated aggressiveness and DNA repair of triple-negative breast cancer. Cell Death Dis. 14, 53. 10.1038/s41419-023-05576-8 36681663 PMC9867751

[B193] Sudan S. K. Deshmukh S. K. Poosarla T. Holliday N. P. Dyess D. L. Singh A. P. (2020). Resistin: an inflammatory cytokine with multi-faceted roles in cancer. Biochim. Biophys. Acta Rev. Cancer 1874, 188419. 10.1016/j.bbcan.2020.188419 32822824 PMC8117252

[B194] Surmacz E. Burgaud J. L. (1995). Overexpression of insulin receptor substrate 1 (IRS-1) in the human breast cancer cell line MCF-7 induces loss of estrogen requirements for growth and transformation. Clin. Cancer Res. 1, 1429–1436. 9815941

[B195] Tang S.-Y. Xie H. Yuan L.-Q. Luo X.-H. Huang J. Cui R.-R. (2007). Apelin stimulates proliferation and suppresses apoptosis of mouse osteoblastic cell line MC3T3-E1 via JNK and PI3-K/Akt signaling pathways. Peptides 28, 708–718. 10.1016/j.peptides.2006.10.005 17109997

[B196] Teras L. R. Patel A. V. Wang M. Yaun S.-S. Anderson K. Brathwaite R. (2020). Sustained weight loss and risk of breast cancer in women 50 Years and older: a pooled analysis of prospective data. J. Natl. Cancer Inst. 112, 929–937. 10.1093/jnci/djz226 31845728 PMC7492760

[B197] Thiagarajan P. S. Zheng Q. Bhagrath M. Mulkearns-Hubert E. E. Myers M. G. Lathia J. D. (2017). STAT3 activation by leptin receptor is essential for TNBC stem cell maintenance. Endocr. Relat. Cancer 24, 415–426. 10.1530/ERC-16-0349 28729467 PMC5551450

[B198] Treeck O. Buechler C. Ortmann O. (2019). Chemerin and cancer. Int. J. Mol. Sci. 20, 3750. 10.3390/ijms20153750 31370263 PMC6695761

[B199] Tulotta C. Ottewell P. (2018). The role of IL-1B in breast cancer bone metastasis. Endocr. Relat. Cancer 25, R421–R434. 10.1530/ERC-17-0309 29760166 PMC5987176

[B327] Tyagi A. Sharma S. Wu K. Wu S.-Y. Xing F. Liu Y. (2021). Nicotine promotes breast cancer metastasis by stimulating N2 neutrophils and generating pre-metastatic niche in lung. Nat. Commun. 12, 474. 10.1038/s41467-020-20733-9 33473115 PMC7817836

[B200] Uribesalgo I. Hoffmann D. Zhang Y. Kavirayani A. Lazovic J. Berta J. (2019). Apelin inhibition prevents resistance and metastasis associated with anti-angiogenic therapy. EMBO Mol. Med. 11, e9266. 10.15252/emmm.201809266 31267692 PMC6685079

[B201] Vasiukov G. Novitskaya T. Zijlstra A. Owens P. Ye F. Zhao Z. (2020). Myeloid cell-derived TGFβ signaling regulates ECM deposition in mammary carcinoma via adenosine-dependent mechanisms. Cancer Res. 80, 2628–2638. 10.1158/0008-5472.CAN-19-3954 32312837 PMC7299805

[B202] Vazquez Rodriguez G. Abrahamsson A. Jensen L. D. E. Dabrosin C. (2018). Adipocytes promote early steps of breast cancer cell dissemination via interleukin-8. Front. Immunol. 9, 1767. 10.3389/fimmu.2018.01767 30105032 PMC6077262

[B203] Villodre E. S. Hu X. Larson R. Finetti P. Gomez K. Balema W. (2021). Lipocalin 2 promotes inflammatory breast cancer tumorigenesis and skin invasion. Mol. Oncol. 15, 2752–2765. 10.1002/1878-0261.13074 34342930 PMC8486564

[B204] Voudouri K. Berdiaki A. Tzardi M. Tzanakakis G. N. Nikitovic D. (2015). Insulin-like growth factor and epidermal growth factor signaling in breast cancer cell growth: focus on endocrine resistant disease. Anal. Cell Pathol. (Amst) 2015, 975495. 10.1155/2015/975495 26258011 PMC4518167

[B205] Wang C.-H. Wang P.-J. Hsieh Y.-C. Lo S. Lee Y.-C. Chen Y.-C. (2018a). Resistin facilitates breast cancer progression via TLR4-mediated induction of mesenchymal phenotypes and stemness properties. Oncogene 37, 589–600. 10.1038/onc.2017.357 28991224

[B206] Wang F. Gao S. Chen F. Fu Z. Yin H. Lu X. (2014). Mammary fat of breast cancer: gene expression profiling and functional characterization. PLoS One 9, e109742. 10.1371/journal.pone.0109742 25291184 PMC4188628

[B207] Wang L. Li H. Wang J. Gao W. Lin Y. Jin W. (2011). C/EBP ζ targets to neutrophil gelatinase-associated lipocalin (NGAL) as a repressor for metastasis of MDA-MB-231 cells. Biochim. Biophys. Acta 1813, 1803–1813. 10.1016/j.bbamcr.2011.06.010 21741997

[B208] Wang L. Tang C. Cao H. Li K. Pang X. Zhong L. (2015). Activation of IL-8 via PI3K/Akt-dependent pathway is involved in leptin-mediated epithelial-mesenchymal transition in human breast cancer cells. Cancer Biol. Ther. 16, 1220–1230. 10.1080/15384047.2015.1056409 26121010 PMC4622725

[B209] Wang S. Su X. Xu M. Xiao X. Li X. Li H. (2019). Exosomes secreted by mesenchymal stromal/stem cell-derived adipocytes promote breast cancer cell growth via activation of Hippo signaling pathway. Stem Cell Res. Ther. 10, 117. 10.1186/s13287-019-1220-2 30971292 PMC6458638

[B210] Wang T. Fahrmann J. F. Lee H. Li Y.-J. Tripathi S. C. Yue C. (2018b). JAK/STAT3-Regulated fatty acid β-oxidation is critical for breast cancer stem cell self-renewal and chemoresistance. Cell Metab. 27, 136–150. 10.1016/j.cmet.2017.11.001 29249690 PMC5777338

[B211] Wang Y. Lam J. B. Lam K. S. L. Liu J. Lam M. C. Hoo R. L. C. (2006). Adiponectin modulates the glycogen synthase kinase-3beta/beta-catenin signaling pathway and attenuates mammary tumorigenesis of MDA-MB-231 cells in nude mice. Cancer Res. 66, 11462–11470. 10.1158/0008-5472.CAN-06-1969 17145894

[B212] Wang Y.-Y. Chen H.-D. Lo S. Chen Y.-K. Huang Y.-C. Hu S. C.-S. (2020). Visfatin enhances breast cancer progression through CXCL1 induction in tumor-associated macrophages. Cancers (Basel) 12, 3526. 10.3390/cancers12123526 33256011 PMC7760195

[B213] Wang Y.-Y. Hung A. C. Lo S. Yuan S.-S. F. (2021). Adipocytokines visfatin and resistin in breast cancer: clinical relevance, biological mechanisms, and therapeutic potential. Cancer Lett. 498, 229–239. 10.1016/j.canlet.2020.10.045 33152400

[B214] Wang Y.-Y. Lehuédé C. Laurent V. Dirat B. Dauvillier S. Bochet L. (2012). Adipose tissue and breast epithelial cells: a dangerous dynamic duo in breast cancer. Cancer Lett. 324, 142–151. 10.1016/j.canlet.2012.05.019 22643115

[B215] Ward Z. J. Bleich S. N. Cradock A. L. Barrett J. L. Giles C. M. Flax C. (2019). Projected U.S. State-level prevalence of adult obesity and severe obesity. N. Engl. J. Med. 381, 2440–2450. 10.1056/NEJMsa1909301 31851800

[B216] Watts N. B. (1990). Individualized goal setting for diabetic control. Diabetes Care 13, 811–812. 10.2337/diacare.13.7.811 2387200

[B217] Wei L. Li K. Pang X. Guo B. Su M. Huang Y. (2016). Leptin promotes epithelial-mesenchymal transition of breast cancer via the upregulation of pyruvate kinase M2. J. Exp. Clin. Cancer Res. 35, 166. 10.1186/s13046-016-0446-4 27769315 PMC5073421

[B218] Wenners A. S. Mehta K. Loibl S. Park H. Mueller B. Arnold N. (2012). Neutrophil gelatinase-associated lipocalin (NGAL) predicts response to neoadjuvant chemotherapy and clinical outcome in primary human breast cancer. PLoS One 7, e45826. 10.1371/journal.pone.0045826 23056218 PMC3467272

[B219] Witsch E. Sela M. Yarden Y. (2010). Roles for growth factors in cancer progression. Physiol. (Bethesda) 25, 85–101. 10.1152/physiol.00045.2009 PMC3062054 20430953

[B220] Witzel I. Milde-Langosch K. Schmidt M. Karn T. Becker S. Wirtz R. (2014). Role of urokinase plasminogen activator and plasminogen activator inhibitor mRNA expression as prognostic factors in molecular subtypes of breast cancer. Onco Targets Ther. 7, 2205–2213. 10.2147/OTT.S65344 25506225 PMC4259258

[B221] Wu Q. Li B. Li J. Sun S. Yuan J. Sun S. (2021). Cancer-associated adipocytes as immunomodulators in cancer. Biomark. Res. 9, 2. 10.1186/s40364-020-00257-6 33413697 PMC7792018

[B222] Wu Q. Li B. Li Z. Li J. Sun S. Sun S. (2019). Cancer-associated adipocytes: key players in breast cancer progression. J. Hematol. Oncol. 12, 95. 10.1186/s13045-019-0778-6 31500658 PMC6734503

[B223] Xuan Q. Wang J. Nanding A. Wang Z. Liu H. Lian X. (2014). Tumor-associated macrophages are correlated with tamoxifen resistance in the postmenopausal breast cancer patients. Pathol. Oncol. Res. 20, 619–624. 10.1007/s12253-013-9740-z 24414992

[B224] Yamaguchi J. Ohtani H. Nakamura K. Shimokawa I. Kanematsu T. (2008). Prognostic impact of marginal adipose tissue invasion in ductal carcinoma of the breast. Am. J. Clin. Pathol. 130, 382–388. 10.1309/MX6KKA1UNJ1YG8VN 18701411

[B225] Yan L. Borregaard N. Kjeldsen L. Moses M. A. (2001). The high molecular weight urinary matrix metalloproteinase (MMP) activity is a complex of gelatinase B/MMP-9 and neutrophil gelatinase-associated lipocalin (NGAL). Modulation of MMP-9 activity by NGAL. J. Biol. Chem. 276, 37258–37265. 10.1074/jbc.M106089200 11486009

[B226] Yan Q.-W. Yang Q. Mody N. Graham T. E. Hsu C.-H. Xu Z. (2007). The adipokine lipocalin 2 is regulated by obesity and promotes insulin resistance. Diabetes 56, 2533–2540. 10.2337/db07-0007 17639021

[B227] Yang J. Bielenberg D. R. Rodig S. J. Doiron R. Clifton M. C. Kung A. L. (2009). Lipocalin 2 promotes breast cancer progression. Proc. Natl. Acad. Sci. U. S. A. 106, 3913–3918. 10.1073/pnas.0810617106 19237579 PMC2656179

[B228] Yao H. He S. (2021). Multi-faceted role of cancer-associated adipocytes in the tumor microenvironment (Review). Mol. Med. Rep. 24, 866. 10.3892/mmr.2021.12506 34676881 PMC8554381

[B229] Yee L. D. Mortimer J. E. Natarajan R. Dietze E. C. Seewaldt V. L. (2020). Metabolic health, insulin, and breast cancer: why oncologists should care about insulin. Front. Endocrinol. (Lausanne) 11, 58. 10.3389/fendo.2020.00058 32153503 PMC7045050

[B230] Yeh W.-L. Tsai C.-F. Chen D.-R. (2017). Peri-foci adipose-derived stem cells promote chemoresistance in breast cancer. Stem Cell Res. Ther. 8, 177. 10.1186/s13287-017-0630-2 28750689 PMC5532814

[B231] Yoon Y. S. Kwon A. R. Lee Y. K. Oh S. W. (2019). Circulating adipokines and risk of obesity related cancers: a systematic review and meta-analysis. Obes. Res. Clin. Pract. 13, 329–339. 10.1016/j.orcp.2019.03.006 31003933

[B232] Yu M. Yang Y. Huang C. Ge L. Xue L. Xiao Z. (2022). Chemerin: a functional adipokine in reproductive health and diseases. Biomedicines 10, 1910. 10.3390/biomedicines10081910 36009457 PMC9406010

[B233] Yuan H.-J. Sun K.-W. Yu K. (2014). Leptin promotes the proliferation and migration of human breast cancer through the extracellular-signal regulated kinase pathway. Mol. Med. Rep. 9, 350–354. 10.3892/mmr.2013.1786 24213635

[B234] Zhang Y. Foncea R. Deis J. A. Guo H. Bernlohr D. A. Chen X. (2014). Lipocalin 2 expression and secretion is highly regulated by metabolic stress, cytokines, and nutrients in adipocytes. PLoS One 9, e96997. 10.1371/journal.pone.0096997 24818605 PMC4018437

[B235] Zhao Y. Zheng X. Zheng Y. Chen Y. Fei W. Wang F. (2021). Extracellular matrix: emerging roles and potential therapeutic targets for breast cancer. Front. Oncol. 11, 650453. 10.3389/fonc.2021.650453 33968752 PMC8100244

[B236] Zhu Y. Guo M. Zhang L. Xu T. Wang L. Xu G. (2016). Biomarker triplet NAMPT/VEGF/HER2 as a *de novo* detection panel for the diagnosis and prognosis of human breast cancer. Oncol. Rep. 35, 454–462. 10.3892/or.2015.4391 26531769

